# Highly Stable Garnet Fe_2_Mo_3_O_12_ Cathode Boosts the Lithium–Air Battery Performance Featuring a Polyhedral Framework and Cationic Vacancy Concentrated Surface

**DOI:** 10.1002/advs.202300482

**Published:** 2023-02-20

**Authors:** Yang Qiu, Gaoyang Li, Huimin Zhou, Guoliang Zhang, Liang Guo, Zhanhu Guo, Ruonan Yang, Yuqi Fan, Weiliang Wang, Yong Du, Feng Dang

**Affiliations:** ^1^ Key Laboratory for Liquid–Solid Structural Evolution and Processing of Materials (Ministry of Education) Shandong University Jinan 250061 P. R. China; ^2^ Institute of Environment and Ecology Shandong Normal University Jinan 250358 P. R. China; ^3^ Integrated Composites Lab Department of Mechanical and Construction Engineering Northumbria University Newcastle Upon Tyne NE1 8ST UK; ^4^ School of Environmental and Municipal Engineering Qingdao University of Technology Qingdao 266525 P. R. China; ^5^ State Key Laboratory of Powder Metallurgy Central South University Changsha Changsha 410083 P. R. China

**Keywords:** lithium–air batteries, cathode catalyst, DFT calculations, Fe_2_Mo_3_O_12_, garnet

## Abstract

Lithium–air batteries (LABs), owing to their ultrahigh theoretical energy density, are recognized as one of the next‐generation energy storage techniques. However, it remains a tricky problem to find highly active cathode catalyst operating within ambient air. In this contribution, a highly active Fe_2_Mo_3_O_12_ (FeMoO) garnet cathode catalyst for LABs is reported. The experimental and theoretical analysis demonstrate that the highly stable polyhedral framework, composed of Fe—O octahedrons and M—O tetrahedrons, provides a highly effective air catalytic activity and long‐term stability, and meanwhile keeps good structural stability. The FeMoO electrode delivers a cycle life of over 1800 h by applying a simple half‐sealed condition in ambient air. It is found that surface‐rich Fe vacancy can act as an O_2_ pump to accelerate the catalytic reaction. Furthermore, the FeMoO catalyst exhibits a superior catalytic capability for the decomposition of Li_2_CO_3_. H_2_O in the air can be regarded as the main contribution to the anode corrosion and the deterioration of LAB cells could be attributed to the formation of LiOH·H_2_O at the end of cycling. The present work provides in‐depth insights to understand the catalytic mechanism in air and constitutes a conceptual breakthrough in catalyst design for efficient cell structure in practical LABs.

## Graphical abstract

Garnet type Fe_2_Mo_3_O_12_ cathode catalyst with a highly stable polyhedral framework and efficient capability for the formation and decomposition of Li2O2 and Li2CO3 boosts the performance of Li–air batteries.

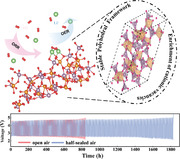

## 1 Introduction

Based on the formation/decomposition of lithium peroxide (Li_2_O_2_), promising lithium–air batteries (LABs) have received tremendous attention as the next generation for energy storage due to their ultrahigh theoretical energy density (3623 Wh kg^−1^), which is competitive to gasoline.^[^ ^1^ ^]^ All the studies reported so far, however, can deliver a satisfactory performance only in a pure O_2_ or simulated air atmosphere. Under the assistance of electrolytes such as ionic liquid‐based electrolytes, and liquid‐based cathode catalysts like InX_3_/MoS_2_, TEMPO/MoP, and LiI/CNT, a well‐constructed LABs cell can show compelling cycle performances.^[^ ^2^ ^]^ It is suggested that the 2D MoS_2_, as a solid cathode catalyst, could deliver a good electrochemical performance in an air‐like atmosphere using an ionic liquid‐based electrolyte and Li_2_CO_3_‐protected Li anode.^[^ ^3^ ^]^ Nevertheless, the presence of H_2_O, CO_2_, and a pretty low content of O_2_ in open air would lead to the formation of various discharge products including low‐ or un‐conductive Li_2_O_2_, Li_2_CO_3_, LiOH, etc.^[^ ^4^ ^]^ Therefore, the cathode catalyst applied in practical LABs in air atmosphere must possess multiple catalytic capabilities for the formation/decomposition of complex discharge products but not only Li_2_O_2_. However, a high‐performance cathode catalyst in the open air without systemic assistance still remains a pivotal challenge for LABs, predominantly owing to limitations in the low catalytic reaction kinetics and chemical selectivity toward multiple discharge products.^[^ ^5^ ^]^

In this work, we reported a conceptual breakthrough for highly active cathode catalysts of LABs. Garnet Fe_2_(MoO_4_)_3_ (FeMoO) was used as a cathode catalyst for LAB. Its high LABs performance only depends on the high catalytic capability in air without the treatments of electrolyte, liquid‐based cathode catalyst, gas selective film, and protective lithium anode. Here, we report a highly active garnet Fe_2_(MoO_4_)_3_ (FeMoO) as a cathode catalyst for LABs operating in the ambient air. Density functional theory (DFT) calculations combined with experimental measurements demonstrated that the highly stable polyhedral framework can boost the catalytic capability in air and maintain the vacancy‐concentrated surface structure during cycling thus leading to impressive electrochemical stability. In addition, a simple half‐sealed testing condition was applied to delay the volatilization of electrolytes and diffusion of H_2_O in the open air, and we found that the death of LABs cells can be mainly attributed to the corrosion of the Li anode, in which no modification has been made. The FeMoO electrode with a cationic vacancy concentrated surface can last for over 1800 h at a low current density of 200 mA g^−1^ without any degradation, and deliver a cycle stability for over 290 cycles at a high current density of 500 mA g^−1^ with a limited capacity of 600 mAh g^−1^ in ambient air. It was revealed that the surface of the Li anode gradually transformed to LiOH during cycling and the formation of LiOH·H_2_O at the end of cycling caused a sudden soar in the charge potential, which results in the death of the LABs. The dominant discharge product is amorphous Li_2−_ *_x_* O_2_, which is preferentially generated on the Mo‐concentrated surface with a high Fe vacancy concentration. The instability of Li_2_O_2_ on Fe vacancy led to the decomposition of the discharge product and can act as an O_2_ pump to accelerate the catalytic reaction in the air. In addition, even though the side product Li_2_CO_3_ is confirmed to be formed through a second reaction between Li_2_O_2_ and CO_2_, the high catalytic capability for the decomposition of Li_2_CO_3_ in the FeMoO electrode is observed and demonstrated.

## 2 Results and Discussion

**Figure 1:**
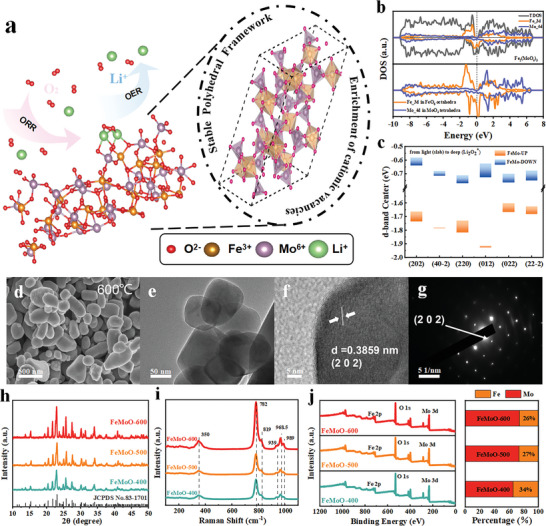
a) Determined unit crystal illustration and schematic illustration of the ORR/OER, illustrating the reaction at the FeMoO; b) Calculated total density of states (DOS) for Fe_2_(MoO_4_)_3_ bulk, Fe 3d in FeO_6_ octahedra, and Mo 4d in MoO_4_ tetrahedra; c) Change in d‐band center of Fe_2_(MoO_4_)_3_ active planes after the adsorption of Li_2_O_2_; d) SEM image, e) TEM image, f) HR‐TEM image, and g) SAED image of FeMoO‐600; h) XRD patterns of the FeMoO samples; i) Raman profiles and j) XPS survey spectra of the FeMoO samples with calculated Fe/Mo ratio.

In this work, we selected Fe_2_(MoO_4_)_3_ with A_3_B_2_(CO_4_)_3_ garnet‐type structure as the cathode catalyst for LABs, in which Fe ions occupy the octahedral B sites and Mo ions occupy the tetrahedral C sites as shown in **Figure** **1**a, while the dodecahedral A sites are unoccupied.^[^ ^6^ ^]^ All the tetrahedra and octahedra are isolated from each other but form into a stable polyhedral frame by corner‐sharing with an oxygen anion. The electron density of state (DOS) of bulk FeMoO garnet in Figure 1b illustrates the half‐metallicity of the FeMoO derived from the Fe spins and stronger electrical conductivity in polyhedral structures, in which the continuous DOS of FeO_6_ octahedra at the Fermi level enhances the electron transport between MoO_4_ tetrahedral nodes. Figure 1c shows the change of the Fe—Mo d‐band center before and after the adsorption of Li_2_O_2_ on the different planes of FeMoO garnet. The up‐spin polarized d‐band center of Fe—Mo on (202) facets (the most stable surface, as discussed in the following DFT calculation) is the most positive with a strong bonding with Li_2_O_2_. The up‐spin polarized d‐band center is still the highest after the initial bonding with Li_2_O_2_, demonstrating the potential catalytic capability in LABs.

The FeMoO garnet catalyst was fabricated using an electrospinning strategy coupled with annealing (Figure S1, Supporting Information). The electrospinning precursors were then sintered at different temperatures (400, 500, and 600 °C) and afforded a batch of three garnet samples designated as FeMoO‐400, FeMoO‐500, and FeMoO‐600, respectively. The field‐emission scanning electron microscopy (FESEM) and transmission electron microscopy (TEM) images in Figure 1d,e and Figure S2, Supporting Information, showed that the FeMoO‐600 sample obtained an irregular morphology with an average size of 500 nm, and the particle size increased from 150 to 500 nm due to the increased temperature for the FeMoO samples. The high‐resolution TEM (HRTEM) image in Figure 1f demonstrated an interlayer spacing of 0.385 nm that corresponds to the (202) plane of the Fe_2_(MoO_4_)_3_ (PDF No. 83‐1701), and the selected area electron diffraction (SAED) in Figure 1g showed a single crystal structure for the FeMoO‐600 particles. The surface areas of the FeMoO‐400, FeMoO‐500, and FeMoO‐600 were 23.8, 10.3, and 8.8 m^2^ g^−1^, respectively, measured by the Brunauer–Emmett–Teller (BET) method as shown in Figure S3, Supporting Information.

From the powder X‐ray diffraction (XRD; Figure 1h) patterns, the diffraction peaks indexed to Fe_2_(MoO_4_)_3_ garnet without any impurity phases for all the samples. The Raman spectra (Figure 1i) exhibited six representative active bands for MoO_4_ tetrahedra in the FeMoO garnet. The peak intensities of the symmetric extension at 968.5 cm^−1^ and asymmetric stretching at 782 cm^−1^ in the MoO_4_ tetrahedron increased with the increase of annealing temperature. The high Raman peak intensity indicates the good polyhedral framework and high occupation ratio of MoO_4_ octahedron site in the garnet structure. X‐ray photoelectron spectroscopy (XPS) was applied to identify the surface condition of the FeMoO catalysts. From the survey XPS spectra (Figure 1j), the Fe/Mo ratio was calculated to be about 2:3 for FeMoO‐400 corresponding to the theoretical molar ratio, and about 1:3 for the FeMoO‐500 and FeMoO‐600 samples, indicating the existence of Fe vacancy. It is revealed that about 30% Fe^3+^ changed into Fe^2+^ (711.5 and 725.1 eV) and few oxygen vacancy (531.3 eV) existed on the surface of the FeMoO particles as observed from the high‐resolution XPS spectra of Mo, Fe, and O (Figure S4, Supporting Information). meanwhile, there was no other valence state and only Mo^6+^ (232.5 and 235.6 eV) was detected for the Mo occupying tetrahedral sites (for a detailed description of the XPS analysis see the Supporting Information).^[^ ^7^ ^]^ It has been demonstrated that oxygen vacancies formed when annealed at reducing atmosphere, and cationic vacancy usually formed at oxidizing atmosphere for oxide particles. The oxidizing atmosphere will lead to the re‐orientation of oxide particles at high temperature and create cationic vacancy on the surface.^[^ ^8^ ^]^ In this work, the FeMoO catalyst was calcined in air, and Mo site maintained the stable Mo^6+^ valance state and the existence of Fe^2+^ was identified by the XPS spectra, furthermore, the Mo/Fe ratio changed to about 4:1 to form a Mo concentrated surface for the FeMoO‐600 sample. As a result, Fe vacancy was created on the surface of FeMoO catalyst. These results indicated that the FeMoO catalyst calcined at a high temperature exhibited high crystallinity as well as a Fe vacancy concentrated surface, which in turn delivered the best performance of LABs.

The electrochemical performance of the FeMoO garnet as potential LABs cathode catalysts are explored using the CR2032‐type coin cells with a lithium plate as the counter electrode. The air electrode consists of FeMoO catalyst, Ketjen Black (KB), and polytetrafluoroethylene (PTFE) binder with a mass ratio of 4:4:2. The average loading mass of the active materials on an air electrode is 1.0 mg cm^−2^. The humidity of the test environment ranged from 10% to 25% as shown in Figure S5, Supporting Information.

**Figure 2:**
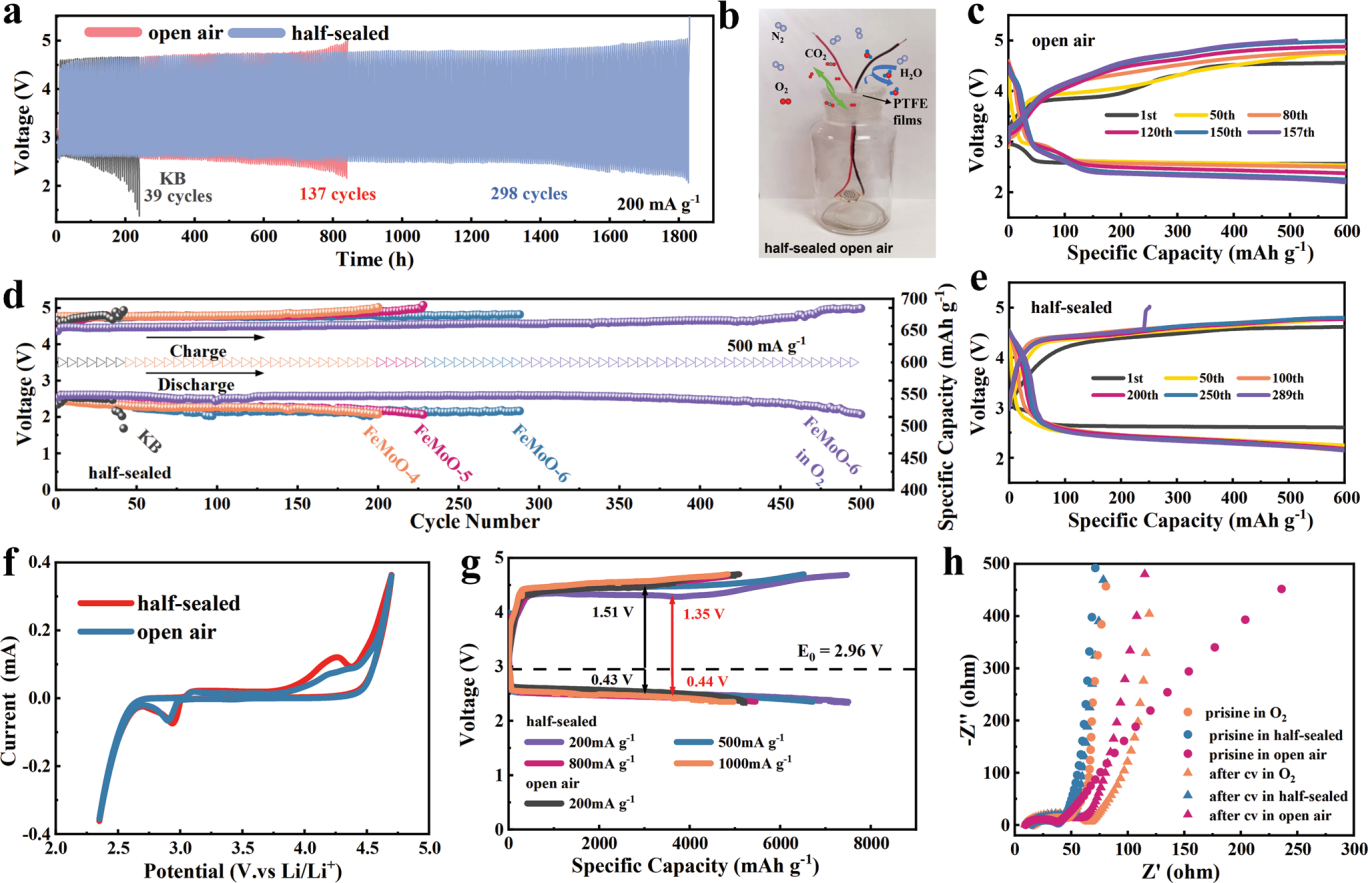
a) Cycling performance of FeMoO‐600 and KB cathode with discharge/charge voltage at 200 mA g^−1^ with a limited capacity of 600 mAh g^−1^ in open air and half‐sealed air condition; b) Photographs of LAB cells in half‐sealed air conditions; Cycling performances of FeMoO‐600 cathode with selected typical discharge/charge profiles at a current density of 500 mA g^−1^ in c) open air and e) half‐sealed air condition; d) cycling performance of KB and FeMoO cathode with terminal voltages at different current density and fixed capacity 600 mAh g^−1^; f) Cyclic voltammetry (CV) curves of FeMoO‐600 cathodes at a scan rate of 0.1 mV s^−1^ with the voltage window of 2.35–4.7 V; g) Rate capability of the FeMoO‐600 electrode; h) EIS curves of the FeMoO‐600 sample both pristine and after CV cycling tests.

In open air, the FeMoO‐600 electrode exhibited superior cycle stability, which can work for over 830 h (137 cycles, **Figure** **2**a, and Figure S6a,b, Supporting Information) at a current density of 200 mA g^−1^ and 157 cycles at 500 mA g^−1^ (Figure 2c and Figure S6a, Supporting Information) with a limited capacity of 600 mAh g^−1^. For comparison, the KB electrode can only last for 38 and 39 cycles at 200 and 500 mA g^−1^ in open air (Figure 2a, and Figure S6a,c,d, Supporting Information). It is worth noting that a sudden increase in the charge potential over 5 V (limited potential of the operating system, LAND, CT 2001A) led to the death of LABs cells at the end of the cycle test (Figure 2c and Figure S6b, Supporting Information). On the other hand, the discharge potential was still maintained at about 2.2 V. It is hypothesized that this sudden increase in the charge potential can be mainly attributed to the corrosion of the Li anode as even slight water moisture in air would contribute to the fast corrosion of the Li anode and thus degrades the LABs cell during cycling.^[^ ^9^ ^]^ Figure S7, Supporting Information, demonstrates this behavior with the direct observation of related XRD patterns on the Li anode as tested in ambient air at a current density of 500 mA g^−1^. The surface of the Li anode changed to LiOH powder with a white color (Figure S7b, Supporting Information) after about 100 h (40 cycles). Furthermore, LiOH·H_2_O was identified (Figure S7b, Supporting Information) after 300 h (125 cycles) and the charge potential increased to 5 V after several cycles. In response, to eliminate or moderate the influence of airflow, a simple half‐sealed LABs working condition was constructed within a restricted space as shown in Figure 2b. The LABs cell was tested in a glass bottle to minimize the possible volatilization of electrolytes in open air and covered by a commercial PTFE film to slow down the diffusion of H_2_O and allow the transportation of components (O_2_, CO_2_, et al.) in air atmosphere. By using the half‐sealed air test condition, the FeMoO‐600 electrode exhibited excellent cycle stability of 298 cycles (1800 h, Figure 2a, and Figure S8a,b, Supporting Information) at 200 mA g^−1^ and 289 cycles (Figure 2d,e) at 500 mA g^−1^ with a limited capacity of 600 mAh g^−1^, which outperformed most of the reported cathode catalyst in LABs. Notably, the FeMoO‐600 electrode delivered steady discharge potentials over 2.5 V for 200 cycles with low overpotentials compared to that in open air, while this electrode was also degraded due to the increased charge potential over 5 V as a result of the Li anode corrosion. On the other hand, as shown in Figure S7a, Supporting Information, the Li anode can still maintain a metal‐like surface in half‐sealed air even after 150 cycles, indicating a slow Li anode corrosion kinetics in a half‐sealed condition. Unfortunately, LiOH·H_2_O can be detected at the end of cycling (690 h, 289 cycles, Figure S7b, Supporting Information), which should be the main reason for the death of LABs cells. It is deduced that the low solubility of LiOH·H_2_O in electrolyte limits the diffusion of Li ion into the electrolyte and leads to the deterioration of LABs cell. For comparison, the FeMoO‐400 and FeMoO‐500 electrodes delivered relatively low cycle performance of 200 and 230 cycles at the current density of 500 mA g^−1^ (Figure 2d and Figure S8d,e, Supporting Information), respectively. Furthermore, the FeMoO‐600 electrode also exhibited excellent electrochemical performance in Li‐oxygen batteries (LOBs), which delivered cycle stability over 500 cycles at 500 mA g^−1^ within a capacity limitation of 600 mAh g^−1^ (Figure 2d and Figure S9, Supporting Information) and can maintain the discharge potential over 2.5 V for 400 cycles. The FeMoO garnet electrode with high crystallinity and cationic vacancy concentrated surface showed the best performance in both LABs and LOBs. In the current contribution, it is the first time to achieve high LABs performance based on the high catalytic activity of the cathode catalyst in ambient air with a large loading mass of 1 mg cm^−2^. In Table S1, Supporting Information, we compared the composition, structure, and loading mass of advanced LABs performances with and without the supports of electrolyte, liquid‐based cathode catalyst, oxygen‐selective membranes (OSM), and protected Li anode. It can be seen that the FeMoO catalyst exhibited outstanding catalytic capability compared to the reported works.

Figure 2f shows the CV curves of the FeMoO‐600 electrode in open and half‐sealed air measured at a scan rate of 0.1 mV s^−1^ within a potential range of 2.35–4.7 V. The coincident onset potentials for the oxygen reduction reaction (ORR) at 2.8 V and the oxygen evolution reaction (OER) at 3.12 and 3.75 V, and the integral areas of the CV curves indicated that the half‐sealed air condition did not influence the catalytic capability and reaction kinetics of the FeMoO electrode compared to those in open air. As shown in Figure 2g, the FeMoO‐600 electrode delivered the discharge/charge capacities of 7503.4/7471.6, 6720.5/6520.3, 5455.2/5100.3, and 4972.5/4845.8 mAh g^−1^ with a cutoff voltage window of 2.35–4.7 V at 200, 500, 800, and 1000 mA g^−1^ in half‐sealed air with the Coulombic efficiencies of 99.57%, 97.02%, 93.49%, and 97.45%, respectively, and the discharge potential of 2.5 V is similar to those in O_2_ (2.55 V, Figure S10, Supporting Information) and open air (2.5 V). Meanwhile, the FeMoO‐600 electrode delivered larger discharge/charge capacities in O_2_ (14 417/13 415 mAh g^−1^ at 200 mA g^−1^, Figure S10a, Supporting Information) and lower discharge/charge capacities in open air (5213/5083 mAh g^−1^ at 200 mA g^−1^, Figure S10b, Supporting Information). The electrochemical impedance spectroscopy (EIS) data were also collected before and after the CV test at the open‐circuit voltage as displayed in Figure 2h. The charge transfer resistance (*R*_ct_) of the FeMoO‐600 electrode changed slightly from the pristine 39 to 52 Ω after CV cycles in half‐sealed air, which is similar to that in O_2_ (from the pristine 44.8 to 66 Ω) and open air (from the pristine 37.7 to 45.2 Ω). These results indicated that there is no influence on the catalytic capacity of the FeMoO catalysts in different air test conditions. The different *R*_ct_ values of the FeMoO electrode tested in air and O_2_ should be attributed to the different composition and morphology of the discharge products.

**Figure 3:**
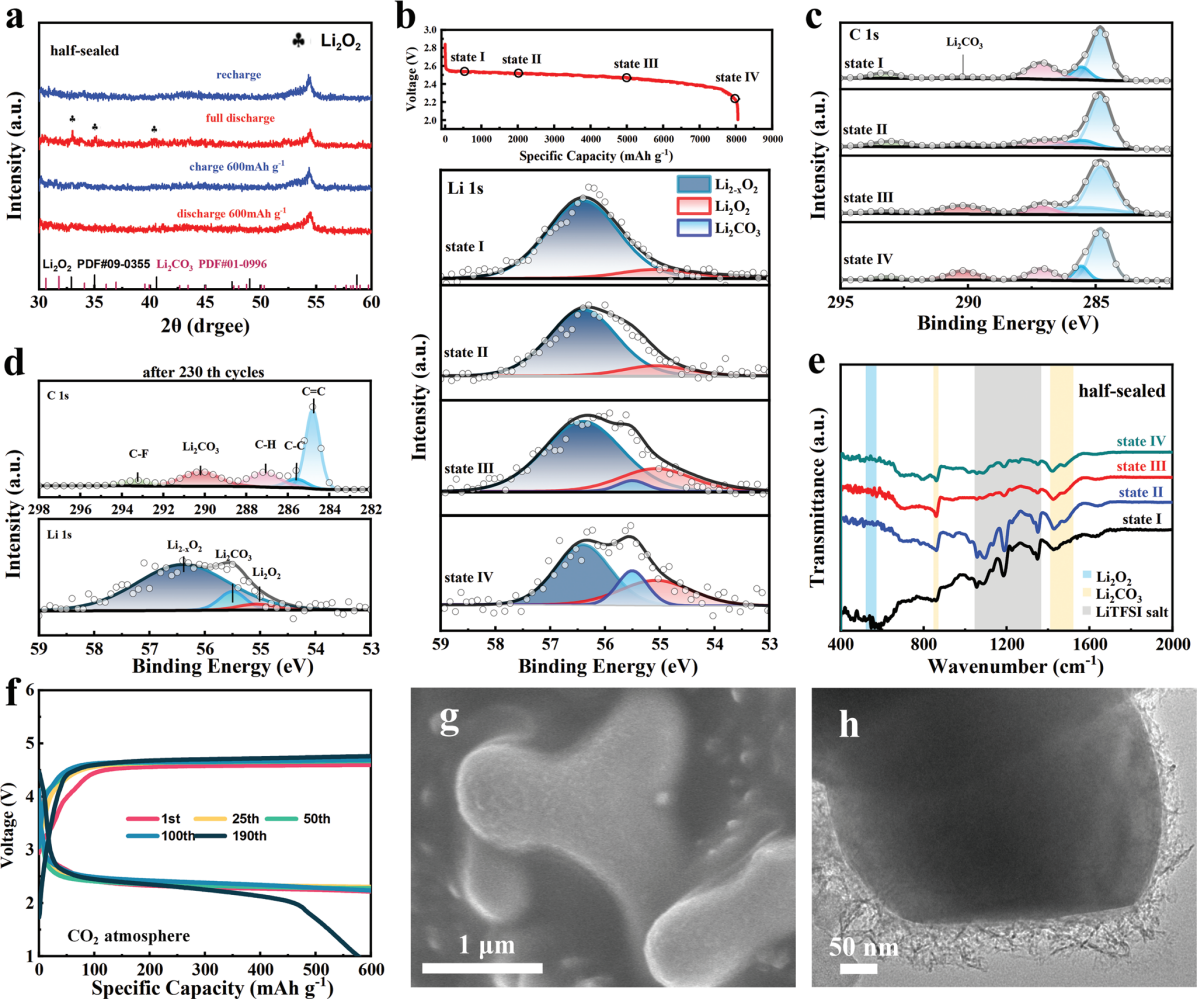
a) XRD patterns of FeMoO‐600 electrodes at different discharge/charge stages with 200 mA g^−1^current density; b) First discharge curve of FeMoO‐600 electrode at a current of 500 mA g^−1^ with an upper capacity of 8000 mAh g^−1^ and high‐resolution XPS spectra of the core level of Li 1s at the corresponding states; c) High‐resolution XPS spectra of C 1s core level of the FeMoO‐600 electrodes at different states corresponding to states (I–IV) in (b), respectively; d) High‐resolution XPS spectra of Li 1s and C 1s core levels of FeMoO‐600 electrodes after the 230th cycle; e) FT‐IR spectra of the FeMoO‐600 electrodes after the first discharge at states corresponding to states (I–IV) in (b), respectively; f) Cycling performances of FeMoO‐600 cathode with selected typical discharge/charge profiles at a current density of 500 mA g^−1^ in CO_2_ condition; g) SEM and h) TEM images of FeMoO‐600 after discharged to 600 mAh g^−1^ at 200 mA g^−1^.

The evolution of the discharge product on the FeMoO electrode was identified through XRD, XPS, and Fourier transform infrared (FT‐IR) measurements. From the XRD pattern in **Figure** **3**a, the discharge products in half‐sealed air were amorphous structures, and no diffraction peaks belonging to Li_2_O_2_ and other possible side products (Li_2_CO_3_ and LiOH) were identified at the initial stage (600 mAh g^−1^). On the contrary, the diffraction peaks of Li_2_O_2_ were identified after full discharge. Furthermore, similar XRD results were obtained for the discharge products in open air and pure O_2_ (Figure S11, Supporting Information). Ex situ XPS spectra in Figure 3b–d and Figure S12, Supporting Information, demonstrated that the main discharge products of the cathode in air were amorphous Li_2−_ *_x_* O_2_ and Li_2_CO_3_ corresponding with the reported works.^[^ ^10^ ^]^ As shown in the high‐resolution XPS spectrum of Li 1s (Figure 3b,d) of the discharge product at different discharge stages, the peak at around 56.4 eV appeared in the Li 1s profiles is associated with the Li_2−_ *_x_* O_2_ (mixture of LiO_2_ and Li_2_O_2_), while the additional peak located at 55.07 eV is attributed to the formation of Li_2_O_2_.^[^ ^11^ ^]^ At the initial discharge stage (600 mAh g^−1^), the discharge products were mainly amorphous Li_2−_ *_x_* O_2_ with few Li_2_O_2_, but no Li_2_CO_3_ and other side products were identified (Figure 3b). After the full discharge process (≈8000 mAh g^−1^), the peak belonging to the Li_2_O_2_ became stronger indicating the conversion from Li_2−_ *_x_* O_2_ to Li_2_O_2_, and the peak of Li_2_CO_3_ (55.5 eV)^[^ ^12^ ^]^ was identified when discharging to 2000 mAh g^−1^. From the high‐resolution XPS spectrum of C 1s (Figure 3c), the peak belonging to Li_2_CO_3_ (290.2 eV)^[^ ^13^ ^]^ can be also observed at the same stage. It is suggested that the formation of Li_2_CO_3_ might be attributed to the second reaction between Li_2_O_2_ and CO_2_ (2Li_2_O_2_ + 2CO_2_ = 2Li_2_CO_3_ + O_2_).^[^ ^14^ ^]^ After recharging, the discharge products can be decomposed completely, meanwhile, the final discharge product before being fully charged was founded to be Li_2−_ *_x_* O_2_ as shown in Figure S13b, Supporting Information. The FT‐IR spectra of the discharge products (Figure 3e) also indicated that the Li_2_CO_3_ formed during the discharging process, and the peak of Li_2_CO_3_ (1450 and 1497 cm^−1^)^[^ ^15^ ^]^ became stronger when discharge to 2000 mAh g^−1^, corresponding with the XPS analysis. Furthermore, when working for 230 cycles at 500 and 600 mAh g^−1^, the discharge products were mainly Li_2−_ *_x_* O_2_ and Li_2_CO_3_ (Figure 3d), indicating an accumulation of Li_2_CO_3_ during cycling. Similar results were obtained for the discharge product tested in open air, at the same time the discharge products were demonstrated to be mainly amorphous Li_2−_ *_x_* O_2_ for the FeMoO electrode in O_2_ (Figures S14–S16, Supporting Information, see the Supporting Information for a detailed description). In addition, the FeMoO electrode also exhibited high catalytic capability for the decomposition of Li_2_CO_3_, which is pivotal for the performance of LABs in air. As shown in Figure 3f and Figure S17, Supporting Information, the FeMoO electrode (1 mg cm^−2^) was assembled into a Li—CO_2_ cell and worked for 190 cycles at a current density of 500 mA g^−1^ with a limited capacity of 600 mAh g^−1^ showing excellent catalytic capability for the decomposition of Li_2_CO_3_. Li_2_CO_3_ is an unexpected main discharge product for the LABs. The formation of Li_2_CO_3_ mainly comes from the decomposition of electrolyte, side reactions between electrolyte and carbon additive, CO_2_ and discharge products.^[^ ^16^ ^]^ The formation of Li_2_CO_3_ will influence the catalytic reaction kinetics, the morphology of discharge products, and the overpotentials of LABs. Through the formation of Li_2_CO_3_, the decomposition of discharge products becomes complicated. Despite the decomposition of Li_2_O_2_, the decomposition of Li_2_CO_3_ can be proceeded through the direct decomposition, C_2_O_6_^2−^ Mediated, O_2_ evolution, CO_3_^2−^ electrooxidation, and carbon involved pathways.^[^ ^17^ ^]^ As a result, the formation of Li_2_CO_3_ is a mainly problem to develop high performance LABs. In this work, the FeMoO catalyst exhibited superior catalytic capability for the formation/decomposition of Li_2_CO_3_, as the Li—CO_2_ cell can stably worked over 190 cycles. The working mechanism for the formation/decomposition of Li_2_CO_3_ on FeMoO catalyst needs more investigation.

The morphology and microstructure of the discharge product on the FeMoO electrode were identified by SEM and TEM observations. Similar film‐like discharge products with a porous structure formed on the FeMoO catalyst in the half‐sealed and open air conditions as shown in Figure 3g and Figure S18, Supporting Information. The TEM image in Figure 3h and Figure S19, Supporting Information, revealed the sub‐crystalline nature of the discharge product and the lattice spacing of 0.22 nm of the nanocrystals indexed to the (102) plane of Li_2_O_2_ (Figure S19a, Supporting Information). In contrast, the discharge product in pure O_2_ was mainly Li_2−_ *_x_* O_2_ film identified from the SEM and TEM observations (Figures S18c,d and S19d,e, Supporting Information). The different morphology of the discharge products in air and O_2_ might be attributed to the formation of Li_2_CO_3_.

The surface stability of the FeMoO electrode during the discharge process and cycling was also characterized by surface‐sensitive XPS as shown in Figures S12–S15, Supporting Information. From the survey spectra (Figures S12a and S13a, Supporting Information), the Fe:Mo ratio (1:3) of the FeMoO‐600 electrode is not changed during the discharge/charge process even after 230 cycles when compared to the initial state (Figure 1j). From the high‐resolution XPS spectra of Fe and Mo (Figures S12b,c and S13d–f, Supporting Information), no pronounced changes can be observed for the valance state of Fe and Mo. These results revealed that the FeMoO garnet structure can provide a highly stable surface condition for the formation/decomposition of discharge products. Even in the O_2_ atmosphere, actually, the stable surface structure of FeMoO garnet was also identified during the ORR/OER process as shown in Figure S15e,f, Supporting Information.

**Figure 4:**
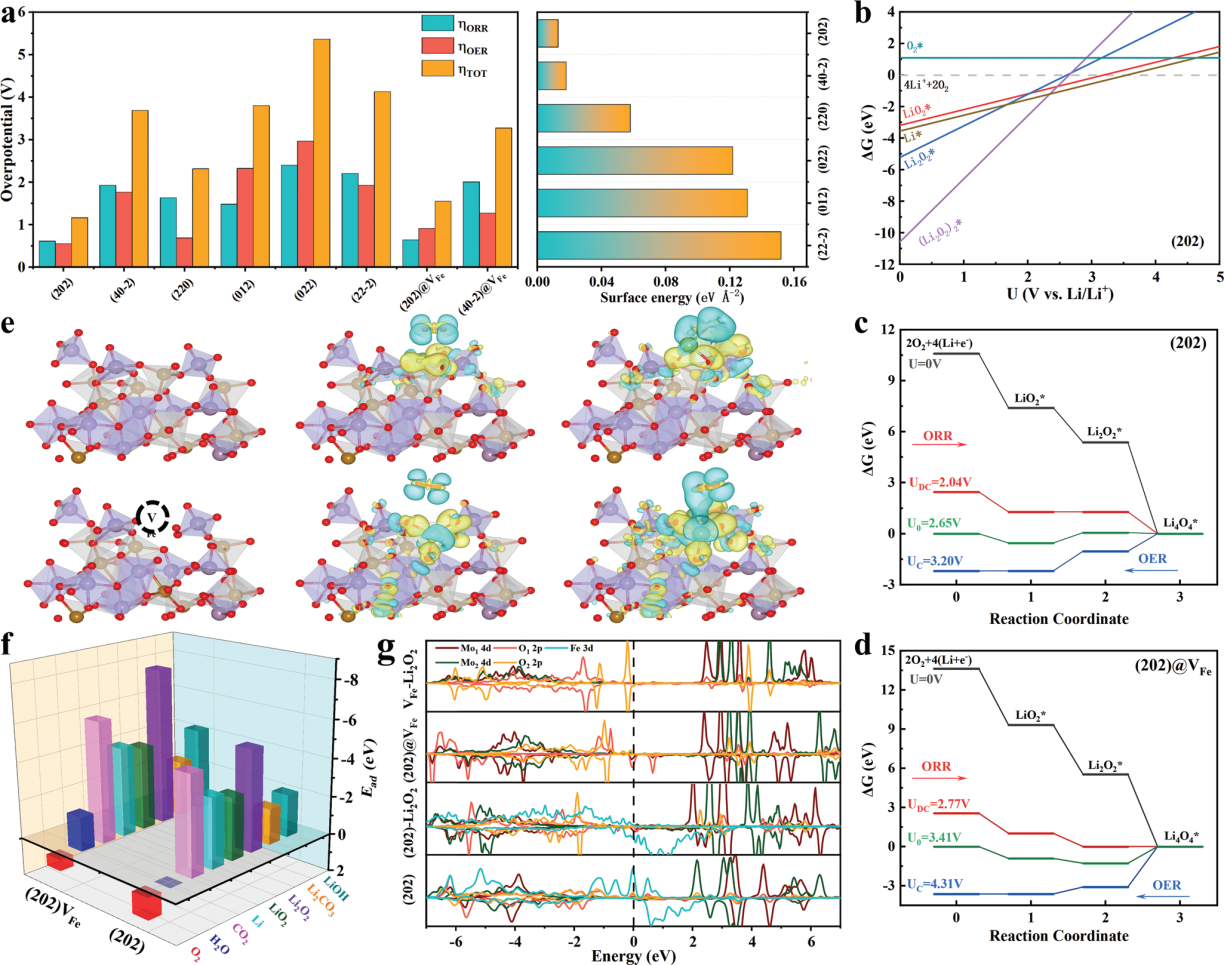
a) Overpotential calculated from energy diagrams and surface energy; b) Potential‐dependent phase diagram of discharge products for (202); Calculated energy diagram of FeMoO; c) (202) facet and d) (202) with Fe vacancy in the ORR and OER processes; e) Charge density difference plots of LiO2* and Li2O2* on (202) plane and (202) with Fe vacancy; f) adsorption energy of different reaction species on (202) plane with and without Fe vacancy of FeMoO; g) PDOS for the Fe and Mo nodes before and after Li2O2 adsorbed on (202) plane and (202) with Fe vacancy.

DFT calculations are applied to gain insights into the catalytic activity of the FeMoO garnet structure. From the PDOS for the Fe and Mo nodes in the bulk FeMoO (Figure 1b), the FeMoO garnet exhibits a strong half‐metallic property, which originates from the spintronic states of the Fe node between the MoO_4_ tetrahedra.^[^ ^18^ ^]^ The good conductivity and stable polyhedral framework can provide stable catalytic capability for long cycle life. We selected six main planes to clarify the catalytic activity of the FeMoO structure with various surface energies, in which (202) as the main lattice plane shows the lowest surface energy as indicated in **Figure** **4**a and Figure S20, Supporting Information. In the phase diagram of the surface species (Figure 4b and Figure S23, Supporting Information), LiO_2_ formed prior at the high potential for all planes compared to Li_2_O_2_ (see the Supporting Information for a detailed description), indicating a single‐electron transfer process during the formation of Li_2_O_2_ on the surface of FeMoO catalyst. As a result, the fundamental three‐step reactions are constructed to evaluate the catalytic capability of the FeMoO garnet for the formation/decomposition of Li_2_O_2_^[^ ^11^, ^19^ ^]^

(1) $$\left({\mathrm{Li}}^{+}+e^{-}\right)+O_{2}⇌{\mathrm{LiO}}_{2}^{*}$$

(2) $${\mathrm{LiO}}_{2}^{*}+\left({\mathrm{Li}}^{+}+e^{-}\right)⇌{\mathrm{Li}}_{2}O_{2}^{*}$$

(3) $${\mathrm{Li}}_{2}O_{2}^{*}+2\left({\mathrm{Li}}^{+}+e^{-}\right)+O_{2}⇌{\mathrm{Li}}_{4}O_{4}^{*}$$

As shown in the free energy diagram of the reaction process (Figure S24 and Table S2, Supporting Information), (022) and (202) planes exhibit the best catalytic capability for the formation/decomposition of Li_2_O_2_ on the Fe active sites between the MoO_4_ tetrahedra, in which the total overpotentials are 1.13 and 1.52 V, respectively. When the discharge products are extended to Li_4_O_4_, the overpotential of the (202) plane decreases to 1.16 V with a low reaction energy barrier for Li_4_O_4_* formation as shown in Figure 4a,c, which is beneficial for the growth as well as decomposition of Li_2_O_2_. Meanwhile, the catalytic capability of the (022) plane decreases significantly with a total overpotential over 4 V, due to the potential control step shift from the generation of LiO_2_* to that of Li_4_O_4_* (Figure S25 and Table S3, Supporting Information).

Figure 4f and Table S4, Supporting Information, show the adsorption energies of the reaction adsorbates on the selected lattice planes of the FeMoO structure. All the calculated surfaces showed high adsorption energy for Li and discharge products, but a weak affinity for O_2_. It is worth noting that the (220), (20−2), (40−2), and (202) are repulsive for O_2_. These results indicated that a refined catalytic reaction can be obtained on the surface of the FeMoO, in which the formation of discharge products will start from the adsorption of Li^+^.^[^ ^18^ ^]^ Notably, the FeMoO structure exhibits extremely high adsorption energies (Figure 4f) for CO_2_ (−5.05 eV) and low adsorption strength for H_2_O (0 eV), indicating the poor reactivity for the formation of LiOH on the FeMoO surface in air. With high adsorption energies for CO_2_, the (202) facet can obtain strong CO_2_ redox activity for Li—CO_2_ batteries. From the charge density difference plots shown in Figure 4e (yellow and blue contours represent the increased and decreased electron density, respectively), the O_2_ part in LiO_2_ is almost dissociated from Li^+^ with little charge transfer when adsorbed on the (202) plane, indicating the unstable adsorption of superoxide species which can avoid the surface oxidation from strong oxidizing intermediate and limit the conversion from LiO_2_ to Li_2_O_2_ on the (202) surface. On the contrary, the Fe nodes of the (202) plane show enhanced chemical binding and charge transfer efficiency with Li_2_O_2._ These results might be associated with the formation of the amorphous Li_2−_ *_x_* O_2_ discharge products identified in experiments, and the high stability of FeMoO garnet catalyst during cycling.

In the experimental sections, the FeMoO‐600 electrode with highly crystalline and Mo‐concentrated surface conditions (Fe vacancy) obtained the best LABs performance. The catalytic capability of the (202) plane with a Fe vacancy is studied in response. After the generation of Fe vacancies, the adsorption energies of the reaction adsorbates exhibit an obvious increase due to a lack of coordination of bridge oxygen between the Fe and Mo node in the MoO_4_ tetrahedra and an increase in unbonded electrons (Figure 4f and Table S4, Supporting Information). The conversion energy gaps from LiO_2_ to Li_2_O_2_ are regulated due to Fe vacancy in the free energy diagram (Figure 4d), resulting in lower total overpotentials of 0.84 and 1.56 V for the formation/decomposition of Li_2_O_2_ and Li_4_O_4_ (Tables S2 and S3, Supporting Information). However, due to the unoccupied cation site and high adsorption energy, LiO_2_ and Li_2_O_2_ are not stable when adsorbed on the Fe vacancy. As shown in Figure 4e, the Fe vacancy site shows a strong elevated surface‐induced dissociation for LiO_2_ and Li_2_O_2_, and releases active O_2_ species, in which the proto‐bridge oxygen as the main bonding site gains most of the charge and the Mo node charge changes very slightly. It is deduced that the instability of Li_2_O_2_ on Fe vacancy site results in the decomposition of discharge product near the tri‐phase interface and leads to the formation of amorphous Li_2−_ *_x_* O_2_ with superior conductivity. On the other hand, the Fe vacancy can act as O_2_ pump during the catalytic reaction process, as the released active O_2_ species can promote the proceeding of the catalytic reaction kinetics, especially in air with a low content of O_2_. As a result, the Mo‐concentrated surface contributed to the excellent LABs performance through altering the structure of discharge products and accelerating the reaction kinetics.

The PDOS after adsorption of Li_2_O_2_ on the (202) plane is shown in Figure 4g to clarify the electronic state during the catalytic process. Compared to the original state, the energy distribution of Mo 4d orbitals at high energy levels becomes less, and the PDOS of the O 3p orbital decreases greatly, indicating that the O in the MoO_4_ tetrahedron near the Fe site plays a critical role in the stable adsorption of the discharge products. Near the Fermi level, the Mo 4d and Fe 3d orbitals become more localized and stronger, especially for the Fe 3d orbitals exhibiting strong polarization, which is beneficial for the charge transfer between discharge products and active sites. Furthermore, as the presence of the Fe vacancy, Mo and the bridge oxygen connecting with the Fe site generate an electron‐rich state and the high‐level electronic states above the Fermi level increase leading to efficiency in adsorption and electron transfer for the reaction species.

## 3 Conclusion

In summary, the FeMoO garnet exhibited highly active catalytic capability as the cathode catalyst in LABs and delivered excellent cycle stability in air. Experimental and theoretical calculations demonstrated that the stable polyhedral framework composed by Fe—O octahedrons and M—O tetrahedrons in the garnet structure provides highly efficient air catalytic activity and stable surface conditions during cycling. We compared the reaction kinetics and electrocatalytic mechanism of the FeMoO garnet in air and pure O_2_. The discharge products were mainly amorphous Li_2−_ *_x_* O_2_ and Li_2_CO_3_ in an air atmosphere and the corrosion of Li anode influenced the cycle stability of the air electrode significantly. The instability nature of LiO_2_ and Li_2_O_2_ on Fe vacancy contributed to the formation of amorphous discharge products and accelerated the catalytic reaction kinetics. These findings revealed the underlying catalytic mechanism of LABs in an air atmosphere to close the knowledge gap between different discharge products derived from complex interface reactions and a facile way to find applicable catalysts in air electrodes. Furthermore, promising as the FeMoO garnet showed a very sound performance in air, this kind of low‐cost metal oxide cathode catalyst could be a potential stock for the commercial engineering design of LABs.

## 4 Experimental Section

### Preparation of the Fe_2_(MoO_4_)_3_

All reagents used in the experiment were analytical grade and without further purification. Fe_2_(MoO_4_)_3_ was prepared via electrospinning as well as the proper calcination method. In brief, 1.5 g polyvinylpyrrolidone (PVP) was dissolved in 10 mL dimethylformamide at room temperature and stirred for 3 h. Meanwhile, 2 mL of acetic acid was added to the solution to adjust the pH. 0.47696 g FeCl_2_⋅4H_2_O and 0.63526 g (NH_4_)_6_Mo_7_O_24_⋅4H_2_O were weighed according to the ratio of Mo/Fe 3:2. The (NH_4_)_6_Mo_7_O_24_⋅4H_2_O was dissolved in 3 mL deionized water. Then the dissolved ammonium molybdate and ferrous chloride were added into the PVP solution, respectively and stir for 10–30 min to get a uniform and transparent red precursor sol. The voltage was set at 13 kV by the electrostatic spinning method and the pushing speed was 0.5 mm min^−1^. The obtained products were collected and dried at 60 °C for 24 h. Finally, they were calcined in the air atmosphere at 2 °C min^−1^ and different temperatures (400, 500, and 600 °C).

### Assembling of LABs

The synthesized ferromolybdate material was used as a catalyst to prepare an oxygen electrode and test the electrochemical properties of the catalyst. The preparation process was as follows. A certain amount of iron molybdate catalyst, KB carbon, and polytetrafluoroethylene (PTFE) binder (4:4:2 by mass) was added to 3 mL of isopropanol to obtain the mixture under ultrasonication. The mixture was uniformly coated on carbon paper and dried under vacuum at 120 °C for 12 h. The loading mass of active materials was 1 mg cm^−2^.

The LAB point cell consists of an anode shell (818 mg, 0.3 mm), prepared oxygen electrode (21 mg, 0.15 mm), glass fiber diaphragm (31 mg, 2.5 mm), lithium metal foil as the anode (45 mg, 0.3 mm), spring plate (364 mg, 0.3 mm), steel sheet (1.528 g, 0.3 mm), negative shell (850 mg, 0.3 mm), and 1 m LiTIFSi/TEGDME as electrolyte. All cells were loaded into an argon‐filled (H_2_O < 0.1 ppm, O_2_ < 0.1 ppm) glove box at room temperature.

### Materials Characterization

XRD (Rigaku D/Max‐r B) was conducted to investigate the phase structure with Cu‐K*α* (*λ* = 1.540 Å) radiation. The morphologies were examined by FESEM (Regulus 8220) and further investigated with HR‐TEM (JEM‐2100F JEOL LTD, 200 kV). Raman spectroscopy was carried out with a Via Reflex instrument with an excitation laser of 532 nm. The specific surface area and pore size distribution were calculated through the N_2_ adsorption–desorption method by BET and Barrett–Joyner–Halenda with Micrometritics (ASAP 2420) apparatus. Simultaneously, high resolution XPS was carried out on an ESCALAB 250Xi spectrometer with an excitation source of Al K*α* = 280.00 eV to detect the surface electronic states and element binding nature. The discharge products accumulated at the anode were characterized by FT‐IR (Nicolet iS 5 FT‐IR). To evaluate the changes in morphology and structure of electrode materials after the electrochemical tests, various characterizations were conducted. All tested cells were disassembled in the glove box after electrochemical measurement and sealed within a polyimide film to minimize the exposure time to the air.

### Electrochemical Measurement

Li–air batteries were tested on a LAND CT2001A multi‐channel battery testing system with a cutoff voltage between 2.35 and 4.7 V (vs Li^+^/Li). The specific capacity and current density were calculated definitely according to the weight of loaded catalysts on carbon paper. The cyclic voltammetry (CV) was measured at a scan rate of 0.1 mV s^−1^ in the potential range of 2.35 to 4.7 V. The EIS was performed within the frequency range from 10^5^ to 0.1 Hz. EIS was performed on a CHI660 electrochemical workstation.

### Theoretical Methods

All data of calculation were obtained by Vienna ab initio simulation package based on DFT.^[^ ^20^ ^]^ The projector augmented wave method was used to describe the electron–ion interaction. The electron exchange and correlation energy were treated by the Perdew–Burke–Ernzerhof function of the generalized gradient approximation.^[^ ^21^ ^]^ The cut‐off energy was set to 400 eV and a k‐point grid was generated by the gamma‐centered Monkhorst–Pack approach.^[^ ^22^ ^]^ The criterion of force relaxation and energy for structural relaxation were 0.02 eV Å^−1^ and 10^−4^ eV, respectively. Evaluation of van der Waals forces with DFT‐D3 dispersion correction for long‐range interactions.^[^ ^23^ ^]^ The vacuum layer was considered to be ≈15 Å on each side. Similar models and parameters were used in catalytic reaction calculations. Gibbs free energy and Bader charge analysis were also successfully applied. Due to large unit cells and low symmetry, all crystal planes calculated were not expanded.^[^ ^24^ ^]^

## Conflict of Interest

The authors declare no conflict of interest.

## Supporting information

Supporting Information

## Data Availability

The data that support the findings of this study are available from the corresponding author upon reasonable request.
