# Supplementary material for: Highly Stable Garnet Fe2Mo3O12 Cathode Boosts the Lithium–Air Battery Performance Featuring a Polyhedral Framework and Cationic Vacancy Concentrated Surface
Source: Adv Sci (Weinh). 2023 Feb 20;10(12):2300482. doi: 10.1002/advs.202300482 (PMC10131855; doi:10.1002/advs.202300482)
Supplement: Supplementary file 1 — Supporting Information [file ADVS-10-2300482-s001.pdf]

Highly stable garnet $Fe_2Mo_3O_{12}$ cathode boosts the Lithium-air battery performance featuring a polyhedral framework and cationic vacancy concentrated surface

Yang Qiu[1,2,#], Gaoyang Li[1,#], Huimin Zhou[1,2], Guoliang Zhang[1], Liang Guo[1], Zhanhu Guo[3], Ruonan Yang[1], Yuqi Fan[2], Weiliang Wang[4,*], Yong Du[5], Feng Dang[1,*]

[1]Key Laboratory for Liquid-Solid Structural Evolution and Processing of Materials (Ministry of Education), Shandong University, Jinan, 250061 China

[2]Institute of Environment and Ecology, Shandong Normal University, Jinan, 250358 China

[3]Integrated Composites Lab, Department of Mechanical and Construction Engineering, Northumbria University, Newcastle Upon Tyne, NE1 8ST, UK

[4]School of Environmental and Municipal Engineering, Qingdao University of Technology, Qingdao 266525, China

[5]State Key Laboratory of Powder Metallurgy Central South University Changsha 410083, China

[*] Corresponding author, dangfeng@sdu.edu.cn

[#] These authors contribute to this work equally.

Table of contents

S1 Materials characterization

Figure S1.The preparation process of FeMoO garnet 4

Figure S2. SEM and TEM images of FeMoO garnet 5

Figure S3. BET surface area and pore size distribution of FeMoO garnet 7

Figure S4. XPS of FeMoO garnet 8

S2 Electrochemical performance

Figure S5. Laboratory relative humidity during test 9

Figure S6. Electrochemical performance of FeMoO electrode in open air 11

Figure S7. Pictures and XRD of Li anode in the open air and half-sealed air condition during cycling 12

Figure S8. Electrochemical performance of FeMoO electrode in half-sealed air condition 13

Figure S9. Electrochemical performance of FeMoO electrode in O_2 14

Figure S10. The rate capability of FeMoO electrode in O_2 and open air 15

S3 Mechanism investigation

Figure S11. XRD of FeMoO electrode at different discharge/charge stages 16

Figure S12. XPS of FeMoO electrode during discharge in half-sealed air condition 17

Figure S13. XPS of FeMoO electrode after recharge in half-sealed air condition 18

Figure S14. XPS of FeMoO electrode during discharge/charge in open air 19

Figure S15. XPS of FeMoO electrode during discharge/charge in O_2 20

Figure S16. FT-IR of FeMoO electrode during discharge in O_2 and open air 22

Figure S17. Electrochemical performance of FeMoO electrode in CO_2 23

Figure S18. SEM image of FeMoO electrode after discharge 24

Figure S19. TEM image of FeMoO electrode after discharge 25

S4 Theoretical calculations

Figure S20. Models of six facets in FeMoO crystal 27

Figure S21. Models of adsorbates on FeMoO different facets 28

Figure S22. Models of adsorbates on two main FeMoO facets 29

Figure S23. Phase diagram of discharge products on FeMoO facets 30

Figure S24. Free energy diagram with Li_2O_2 reaction path on FeMoO facets 32

Figure S25. Free energy diagram with Li_4O_4 reaction path on FeMoO facets 33

Figure S26. Models of adsorbates on FeMoO facets with Fe vacancy 34

Table S1. Comparison of reported Li-air batteries with current work 35

Table S2. Adsorption energy and Bader charge of adsorbates on FeMoO facets 39

Table S3. Calculated overpotential with Li_2O_2 reaction path 40

Table S4. Calculated overpotential with Li_4O_4 reaction path 41

Table S5. The adsorption energy of reaction species on FeMoO facets 42

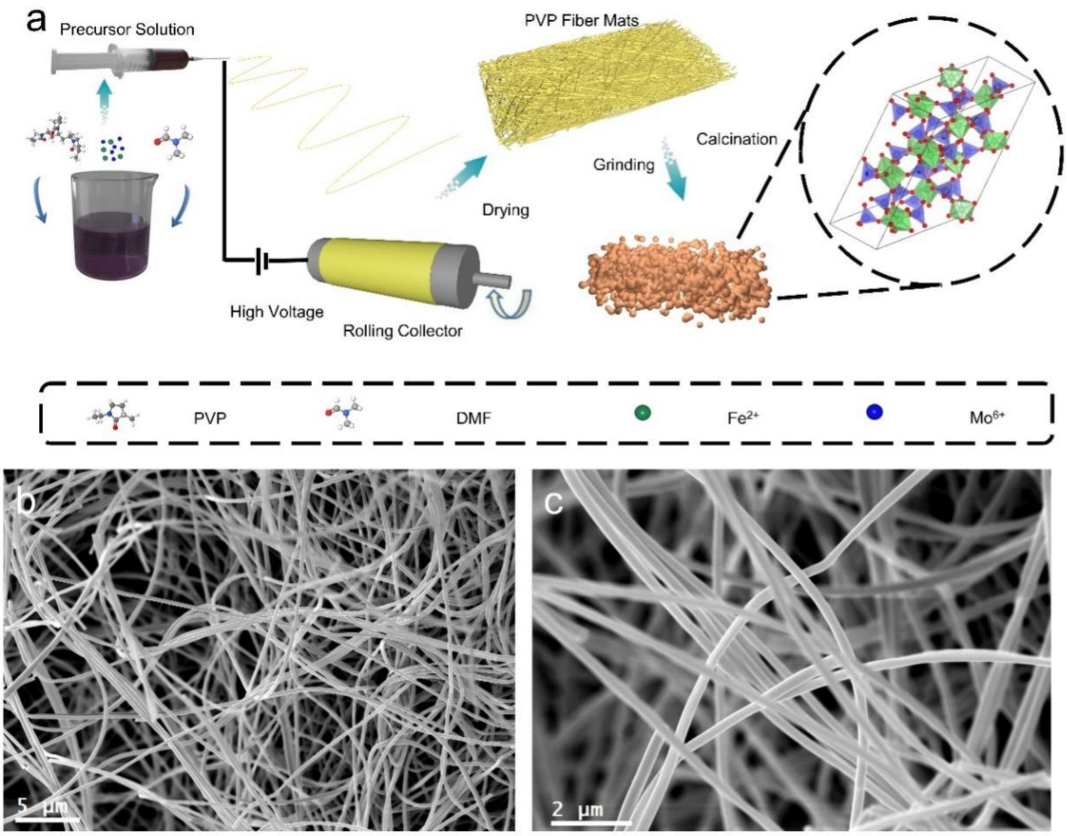

Figure S1 (a) Schematic illustration of synthesis FeMoO garnet through typical electrospinning followed by one-step annealing; (b,c) SEM images of FeMoO precursor prepared by electrospinning.

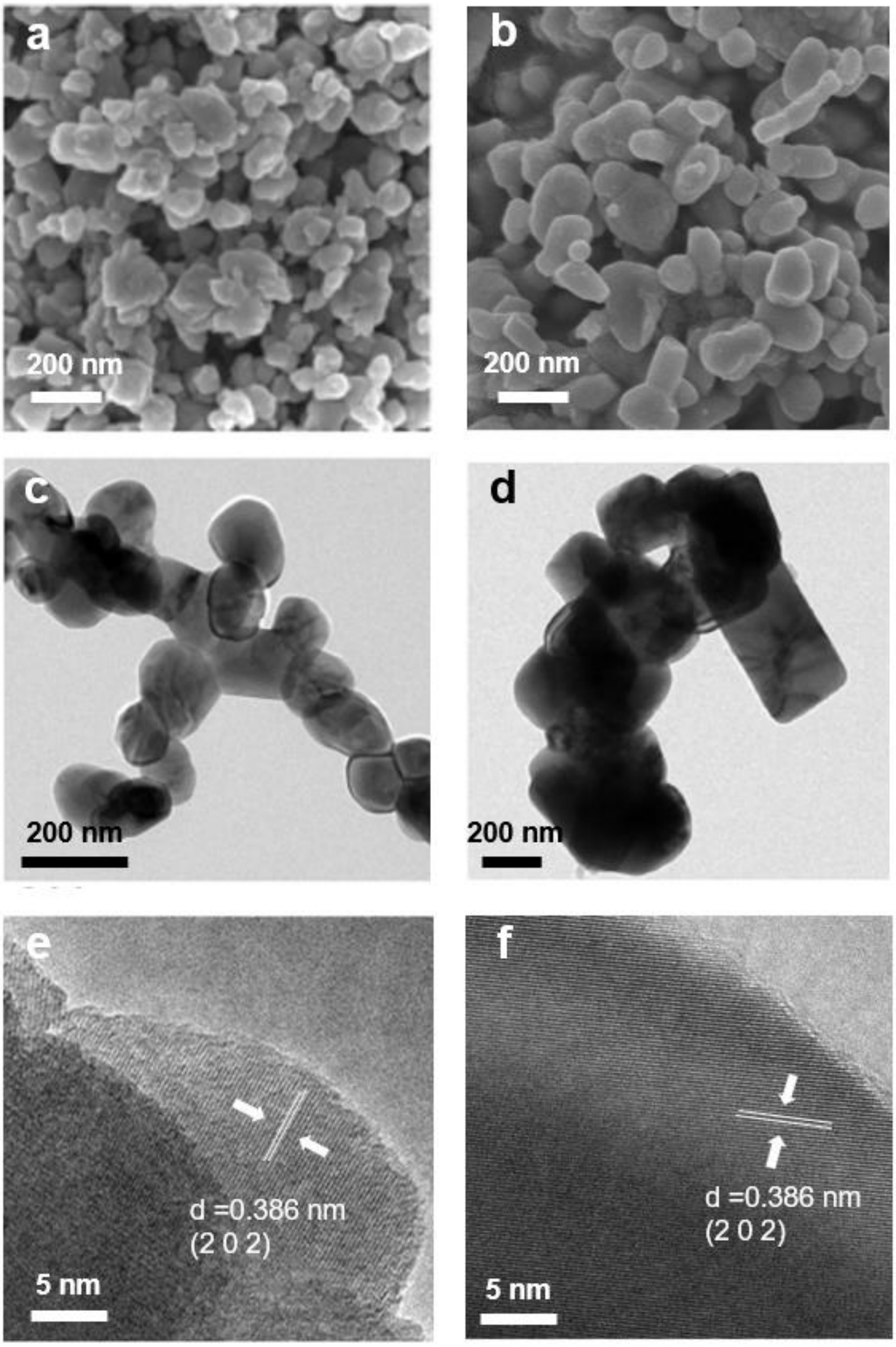

Figure S2 SEM images of (a) FeMoO-400, (b) FeMoO-500; TEM images of (c) FeMoO-400, (d) FeMoO-500; HR-TEM images of (e) FeMoO-400, (f) FeMoO-500.

As shown in Figure 1d and S2a–b, all the FeMoO nanomaterials display an irregular morphology in which the particle size gradually increases along with temperature rising, ca.150 nm for the FeMoO-400 sample (Figure S2a), ca.250 nm for the FeMoO-500 sample (Figure S2b), and ca. 500 nm for FeMoO-600 sample (Figure

1d). TEM images further reveal the morphology of FeMoO nanoparticles (Figure 1e and S2c−d). HRTEM images display the microstructure of FeMoO (Figure 1f and S2e−f) and the lattice spacing of 0.386 nm corresponds to the (202) plane of $Fe_2(MoO_4)_3$ (PDF No. 83-1701), confirming the high crystalline nature of nanoparticles.

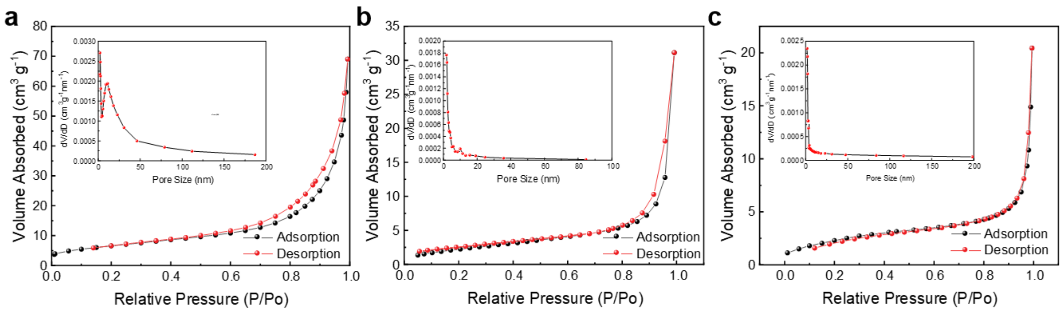

Figure S3 Nitrogen adsorption/desorption isotherms and pore size distribution (inset) of FeMoO-400, FeMoO-500, and FeMoO-600. The specific surface areas calculated by Brunauer-Emmett-Teller are 23.8, 10.3 and 8.8 $m^2\ g^{-1}$ for FeMoO-400, FeMoO-500 and FeMoO-600, respectively.

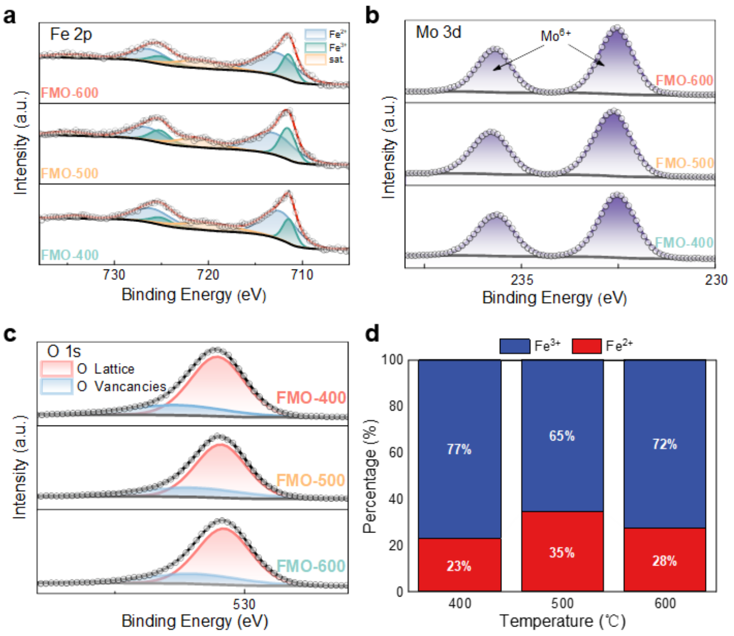

Figure S4 XPS spectra of (a) Fe 2p, (b) Mo 3d, and (c) O 1s; (d) calculated Fe^{3+}/Fe^{2+} ratio for FeMoO samples.

Figure S4 show the electronic valence and chemical composition of FeMoO analyzed by XPS. In Figure S4a, each spin-orbit element of the Fe 2p domain is separated into two main features. The binding energies at 711.5 eV and 725.1 eV are Fe^{2+} oxidation states of $2p_{1/2}$ and $2p_{3/2}$. The two broader peaks at 721.3 eV and 734.8 eV are attributed to the Fe satellite peak, while the Fe^{3+} peak can be split into peaks located at 713 eV and 726.7 eV.[1] The Mo 3d (Figure S4b) is well fitted by two characteristic peaks at 232.5 and 235.6 eV assigned to Mo $3d_{5/2}$ and Mo $3d_{3/2}$, respectively,[2] demonstrating the Mo^{6+} oxidation state. Figure S4c shows the O 1s XPS spectra of 530.5 and 531.4 eV,[3] attributed to the lattice oxygen and vacancy oxygen in the samples, respectively.

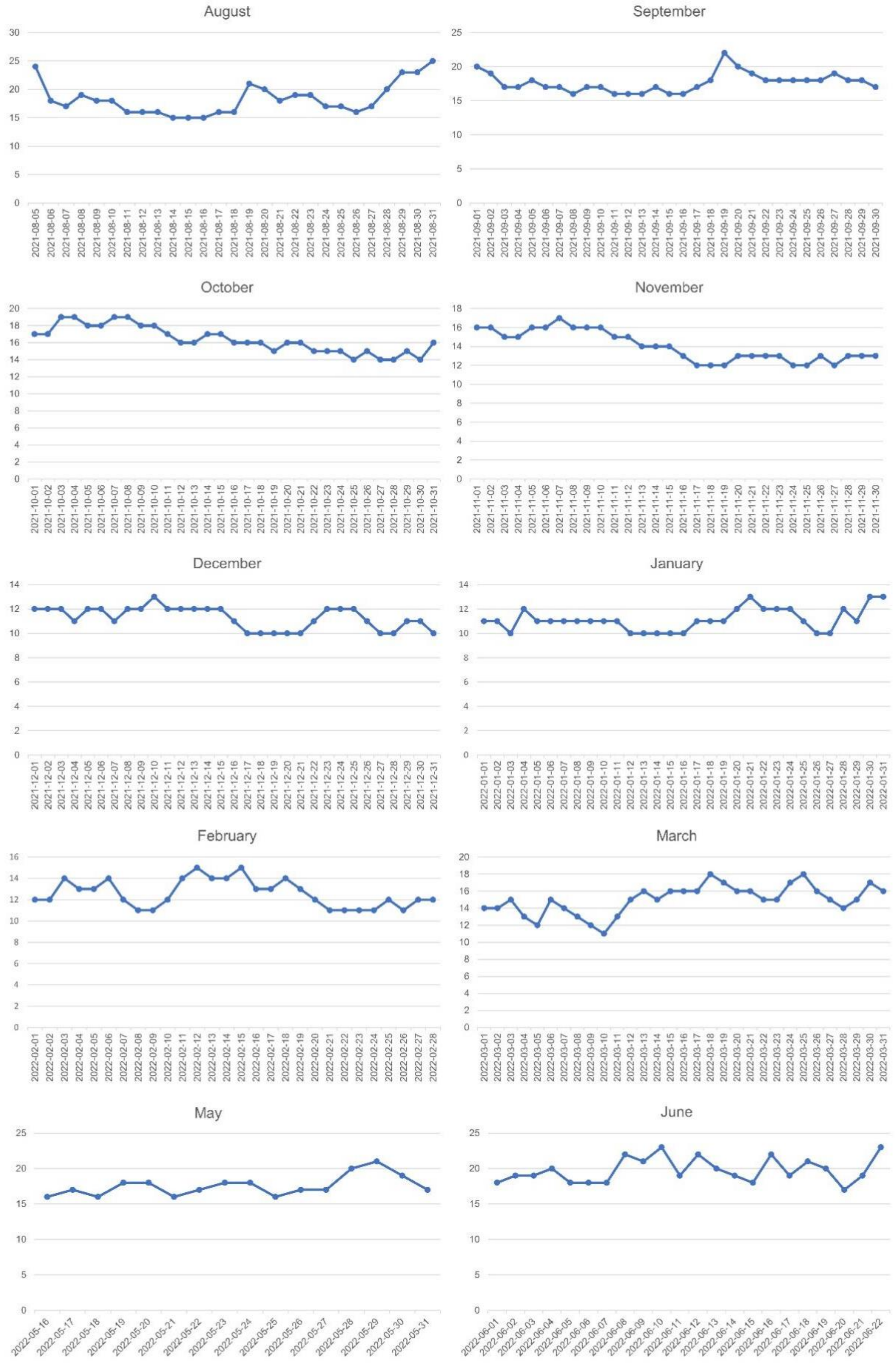

Figure S5 The laboratory relative humidity during the test period.

Figure S5 displays the relative humidity of the laboratory during the lithium-air batteries test period (2021-8-5 to 2022-3-31 and 2022-5-16 to 2022-6-22). All the data were recorded by a hygrometer (Dretec O-230).

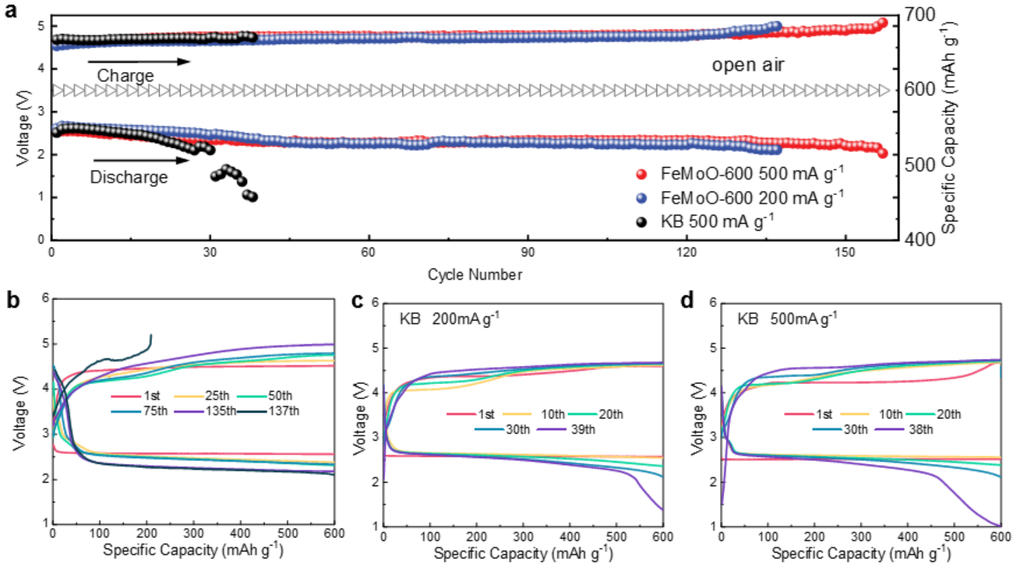

Figure S6 (a) Cycling performance of FeMoO-600 cathode with discharge/charge voltage in the open air; selecting galvanostatic discharge/charge curves of (b) FeMoO-600 and (c) KB cathode at a current density of 200 mA g^{-1} within an upper-limited specific capacity of 600 mAh g^{-1} in the open air; (d) selecting galvanostatic discharge/charge curves of KB cathode at a current density of 500 mA g^{-1} within an upper-limited specific capacity of 600 mAh g^{-1} in the open air.

The cycling stability of the FeMoO-600 electrode was tested in the open air with a capacity of 600 mAh g (Figure 2a, c, and S6a–b). The FeMoO-600 electrode can operate stably for more than 800 hours (137 cycles) at a current density of 200 mA g^{-1} and 400 hours (157 cycles) at a current density of 500 mA g^{-1}. It is worth noting that their discharge voltage was stable above 2.2 V until the charging voltage rise to 5 V to terminate the test. Under the same conditions, the final discharge voltage of the KB electrode is below 2 V after the 30th cycle (Figure S6c and d).

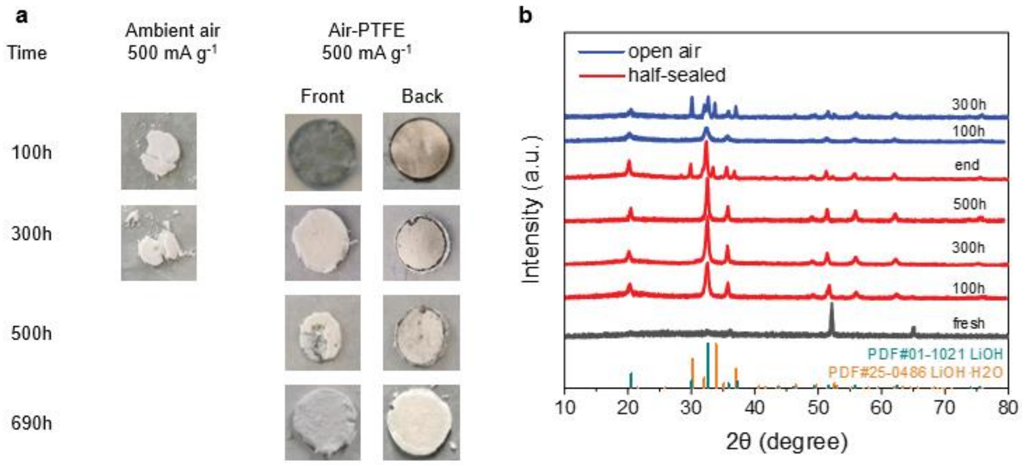

Figure S7 (a) Lithium anode images after running in the open air and half-sealed air condition for different times; (b) XRD patterns of the lithium anodes.

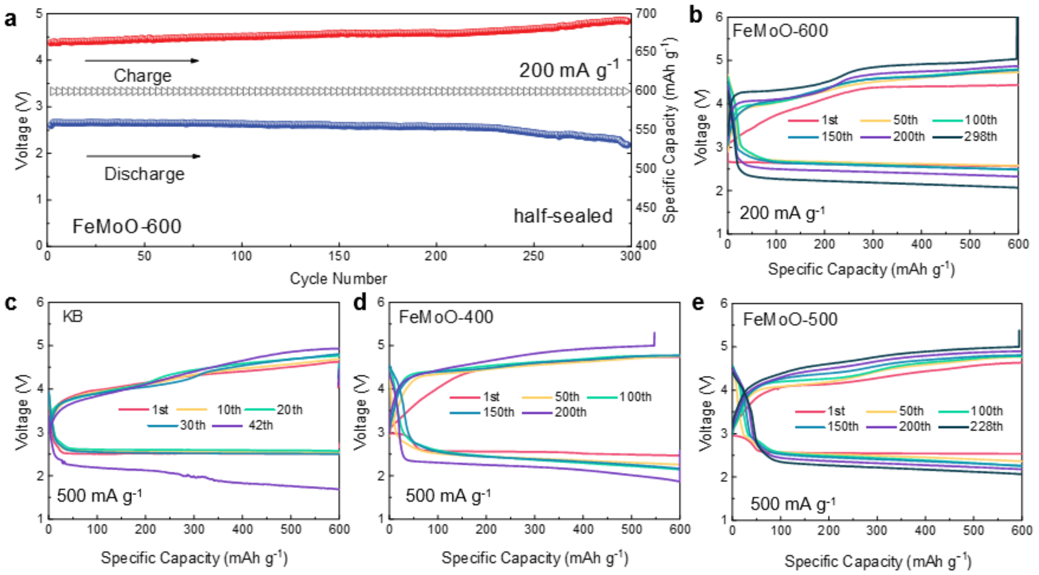

Figure S8 (a) The charge/discharge voltage of FeMoO-600 cathode catalytic cells with a current density of 200 mA g^{-1} in half-sealed air condition; Cycling performance of (b) FeMoO-600, (c) KB, (d) FeMoO-400 and (e) FeMoO-500 electrodes with selected typical galvanostatic discharge/charge profiles and corresponding cycle number.

As in Figure 2a and S8a, the FeMoO-600 electrode exhibited a stable long cycle life of more than 1800 hours (298 cycles) at 200 mA g^{-1} with a limited capacity of 600 mAh g^{-1}. At a high current density of 500 mA g^{-1}, it can still work for 289 cycles while the FeMoO electrode shows a stable discharge plateau with a voltage over 2.35 V (Figure 2e). On the other hand, the KB, FeMoO-400, and FeMoO-500 electrodes show poor performance as shown in Figure S8c–e with the stable operation of 35, 200, and 230 cycles at a current density of 500 mA g^{-1}.

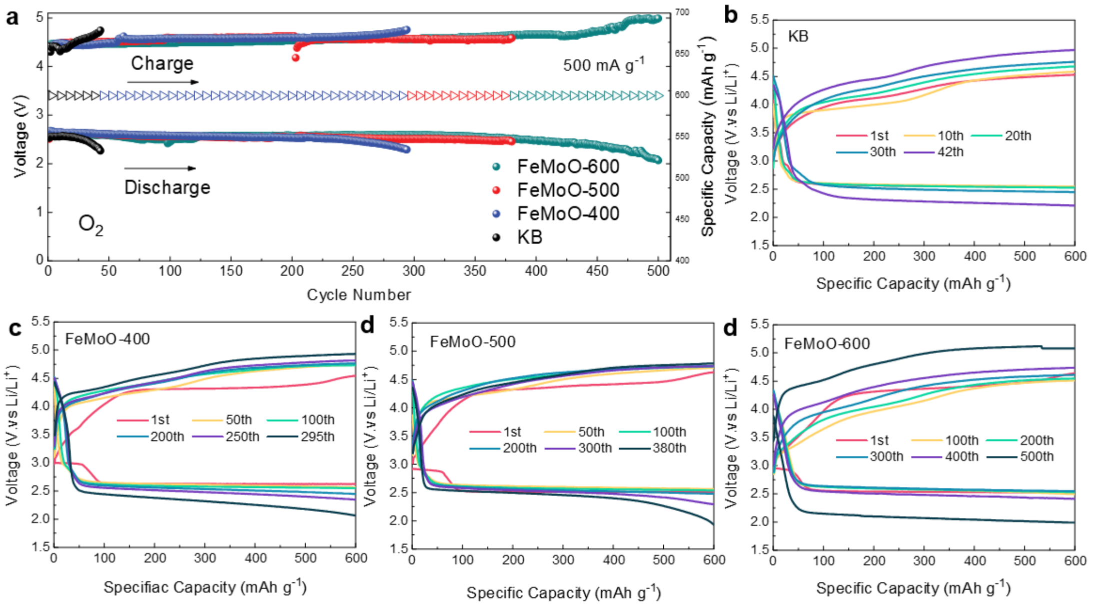

Figure S9 (a) The charge/discharge voltage of FeMoO cathode catalytic cells in O_2 atmosphere, Galvanostatic discharge/charge curves with voltages of (b)KB, (c)FeMoO-400, (c) FeMoO-500 and (d) FeMoO-600 within an upper-limited specific capacity of 600 mAh g^{-1} at a current density of 500 mA g^{-1} in O_2 atmosphere.

As a comparison, the cycle stability of the FeMoO electrode was also tested in a pure oxygen environment (Figure S9a–d). With an upper capacity of 600 mAh g^{-1} and a current density of 500 mA g^{-1}, the FeMoO-600 electrode was able to maintain its capacity for 500 cycles and the discharge voltage was still maintained at 2.5 V in the 400th cycle. FeMoO-500 and FeMoO-400 electrodes can work stably for 380 and 295 cycles, respectively.

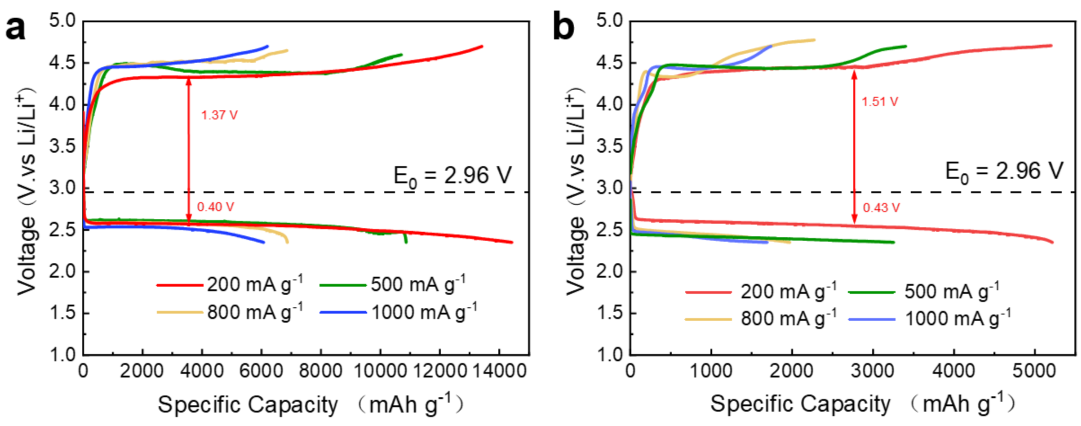

Figure S10 Rate capability of FeMoO-600 electrode in (a) O_2, and (b) open air.

Figure S10a–b displays the rate capability of the FeMoO-600 electrode under different gas atmospheres with voltage window from 2.35 to 4.7 V. In O_2, discharge/charge performances of 14417.2/13415.3, 10865.6/10708.6, 6869.2/6854.9, and 6072.2/6029 mAh g^{-1} were recorded at current densities of 200, 500, 800, and 1000 mA g^{-1} with corresponding Coulomb efficiencies of 93.05%, 98.56%, 99.79%, and 99.29%, respectively.

In the open air, the FeMoO-600 electrode exhibited discharge/charge capacities of 5213.4/5083.4, 3500/3402.9, 1969.5/1717.1, and 1700/1690.5 mAh g^{-1} at 200, 500, 800, and 1000 mA g^{-1}, respectively, with corresponding Coulomb efficiencies of 97.5%, 97.22%, 87.18%, and 99.44%.

.

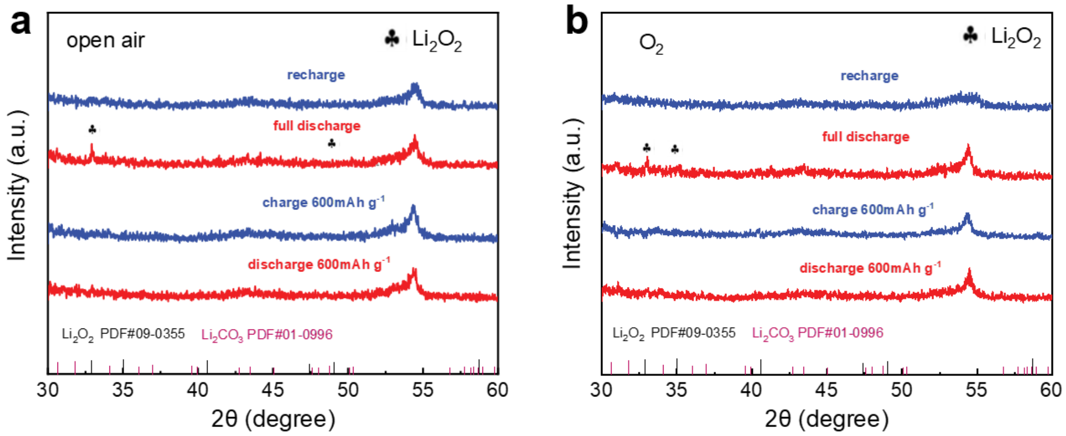

Figure S11 XRD patterns of FeMoO-600 electrode at different discharge/charge stages under 200 mA g^{-1} current densities in (a) open air and (b) O_2 atmosphere.

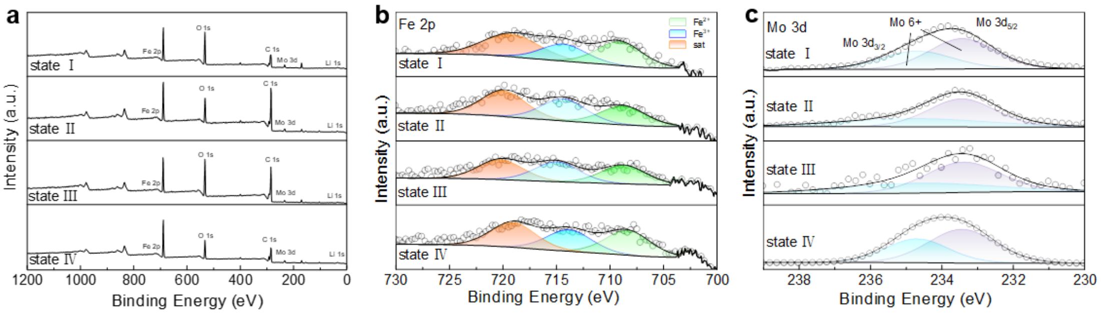

Figure S12 XPS spectra of FeMoO-600 electrode at different stages of 1st electrochemical discharge in half-sealed air condition (a) survey XPS spectra, (b–c) high-resolution XPS spectra of Fe, Mo.

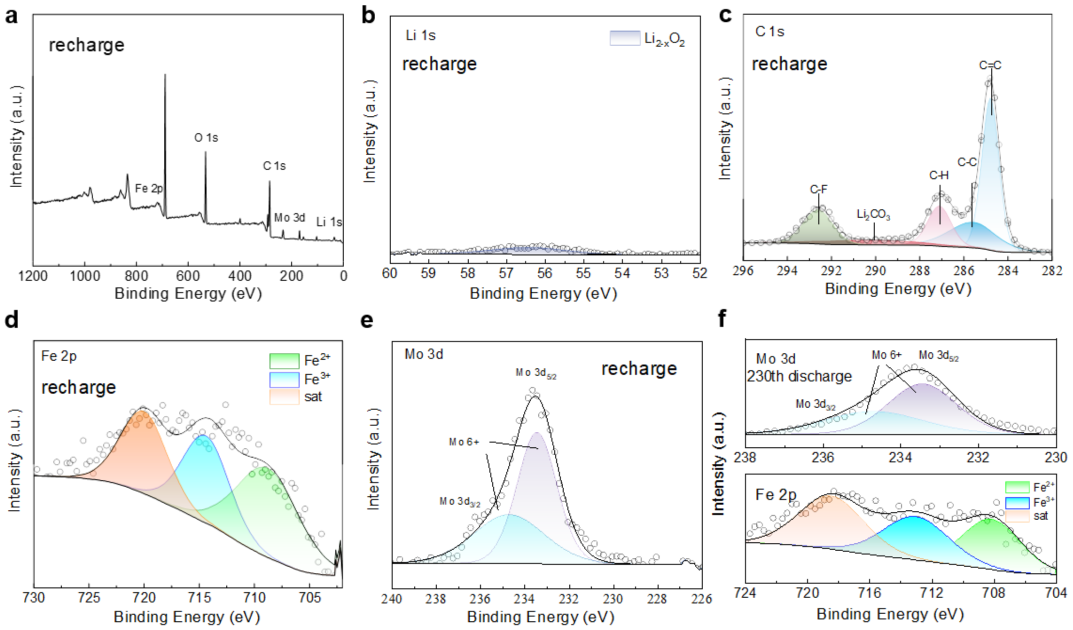

Figure S13 XPS spectra of the charging process in half-sealed air condition: (a) survey XPS spectra (b–e) High-resolution XPS spectra of Li, C, Fe, and Mo after recharging; (f) High-resolution XPS spectra of Fe and Mo after the 230th discharge of FeMoO-600 electrode in half-sealed air condition.

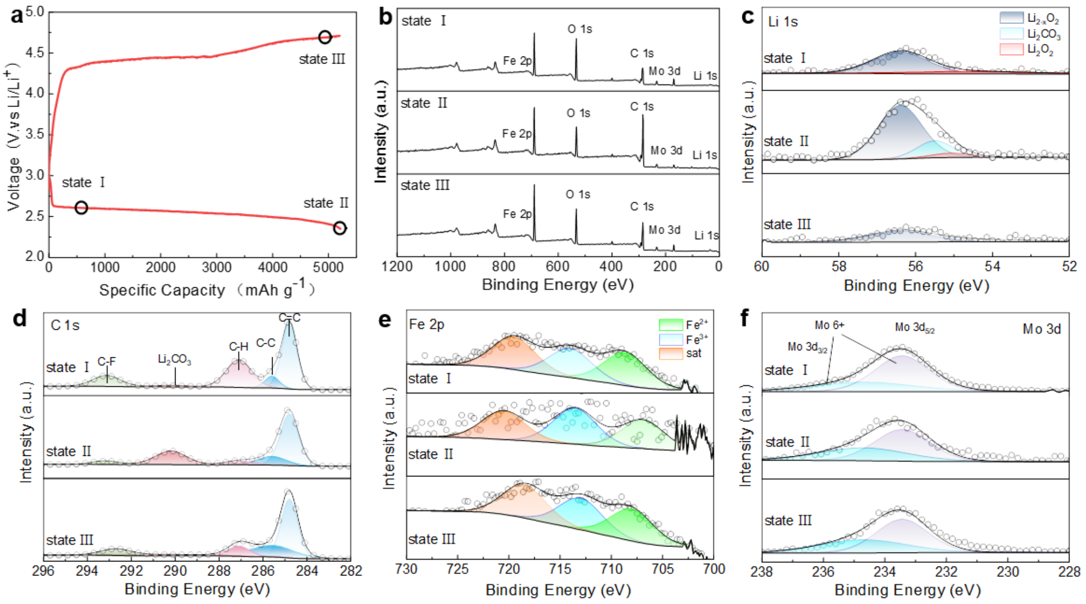

Figure S14 (a) The full discharge/charge curve of the FeMoO-600 electrode in the open air. XPS spectra of FeMoO-600 electrode at different stages: (b) survey XPS spectra, and (c-f) high resolution XPS spectra of Li, C, Fe, and Mo, respectively.

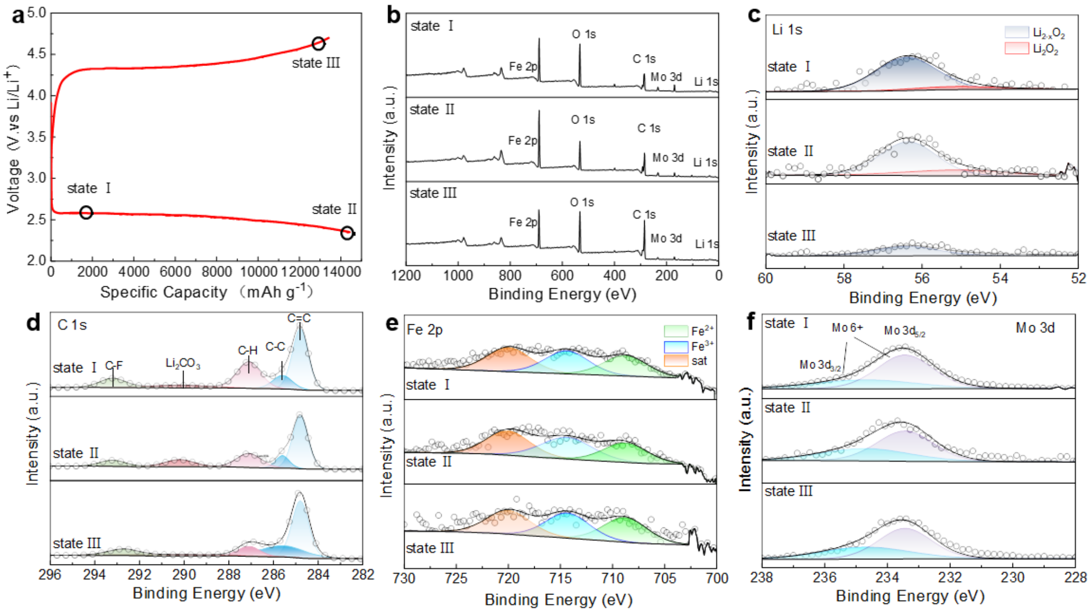

Figure S15 (a) The full discharge/charge curve of the FeMoO-600 electrode in O_2; XPS spectra of FeMoO-600 electrode at different stages: (b) survey XPS spectra, and (c–f) high resolution XPS spectra of Li, C, Fe, and Mo, respectively.

Figure 3b–c and S12–S15 depict the ex-situ high-resolution XPS data of the Li 1s, C1s, Fe 2p, and Mo 3d orbitals on FeMoO-600 electrode at different discharge/charge stages in half-sealed air conditions. The survey spectra (Figure S12a) represent the corresponding elements such as Li, C, Mo, and Fe of the anode at different discharge stages. As shown in Figure 3b, the binding energies of 55.07, 55.5 and 56.4 eV can be assigned to Li_2O_2, Li_2CO_3, and $Li_{2-x}O_2$ (composite of LiO_2 and Li_2O_2).[4] In addition, the peak of Li_2O_2 gradually increases with the discharge process, indicating that part of $Li_{2-x}O_2$ is gradually converted into Li_2O_2. Throughout the discharge process, the low crystallinity mixture $Li_{2-x}O_2$ is always the main product in the discharge process, which is conducive to the improvement of electron transfer and ion migration, and the cycle stability of Li-air batteries.[5] After discharge to 2000 mAh g^{-1}, the peak of Li_2CO_3 is identified and gradually increases with further discharge. After recharging (Figure S13b), the discharge products almost decomposed completely. As shown in Figure 3c,

the C 1s spectrum of the cathode is similar during the discharge process.[6] Among them, the binding energies at 287.1 eV and 293.2 eV can be attributed to the polytetrafluoroethylene (PTFE) binder. The discharge product Li_2CO_3 (290.2 eV) gradually becomes obvious with the discharge process, which is consistent with the Li 1s XPS results (Figure 3b). As shown in Figure S13b, the Fe 2p spectrum is almost unchanged, and the binding energy near the 709 and 714 eV can be attributed to Fe^{2+} and Fe^{3+}. The binding energy near 719.6 eV can be assigned to the satellite peak.[7] As shown in Figure S12c, Mo 3d core level has two peaks at binding energies of 233.4eV and 234.7eV, corresponding with the Mo^{6+} valence state indicating the stable surface condition of FeMoO catalyst in LABs.[8]

In addition, similar results of FeMoO-600 electrode were also obtained testing in open air and oxygen atmosphere (Figure S14−15). Meanwhile, the discharge product was mainly $Li_{2-x}O_2$ for the FeMoO electrode when tested in O_2 and no Li_2CO_3 was detected.

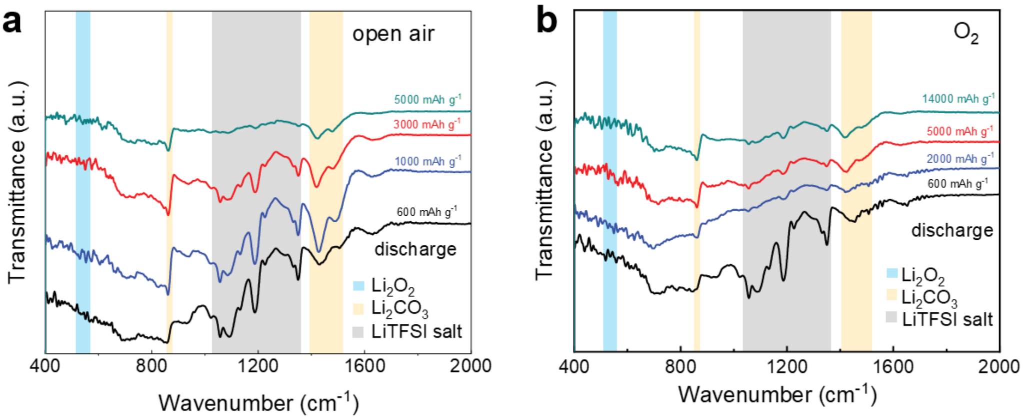

Figure S16 FT-IR spectra of the FeMoO-600 electrode at different capacities during discharge in (a) air and (b) O_2.

Figure S16 shows the FT-IR spectra tested in open air and O_2. The fluctuating peak of Li_2O_2 can be observed at 550cm^{-1}. The peaks at 860 cm^{-1} (C=O), 1450, and 1497 cm^{-1} (*v*CO) can be attributed to low-crystalline Li_2CO_3, which results from the side reaction of carbon and the existence of CO_2 in the air.[9] The LiTFSI/TEGDME electrolyte typical peaks were also detected at 1040 to 1350 cm (*v*SO= 1057 cm^{-1}, *v*SO_2=1134 cm^{-1}, CF = 1186 cm^{-1}, CF = 1226 cm^{-1}, *vas*SO_2 =1334 cm^{-1}, and *vas*SO_2= 1350 cm^{-1}).[10]The peak belonging to Li_2CO_3 became stronger during the discharge process in the open air and was not obviously in O_2. It can be concluded that there existed few Li_2CO_3 as the discharge product in the O_2 atmosphere, and Li_2CO_3 was part of the discharge product in the open air.

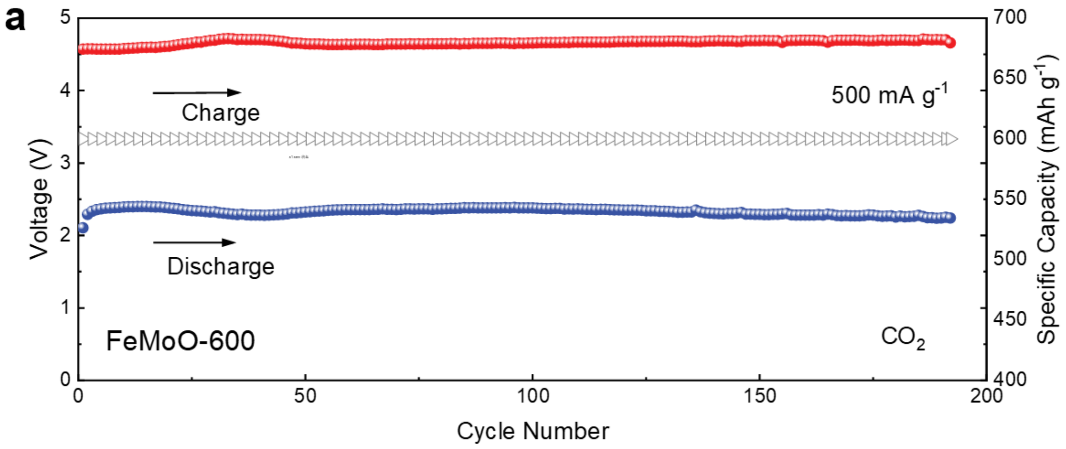

Figure S17 discharge/charge voltage of FeMoO-600 cathode catalytic cells in CO_2 atmosphere at a current density of 500mA g^{-1} and a specific capacity limit of 600mAh g^{-1}.

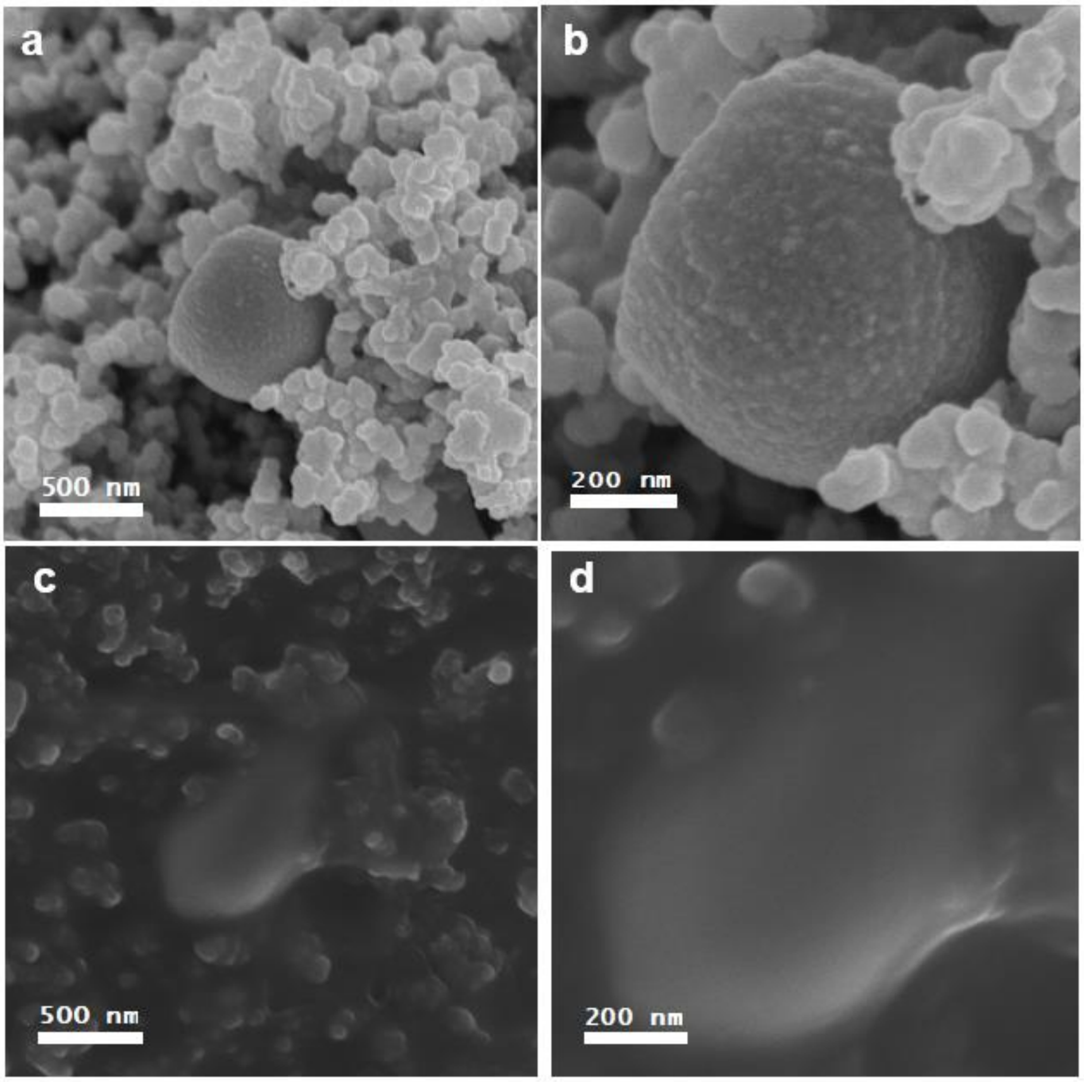

Figure S18 SEM images of discharge products on FeMoO-600 electrode with High- and low-resolution in (a–b) the open air and (c–d) O_2 atmosphere after discharged to 600 mAh g^{-1} at 200 mA g^{-1}.

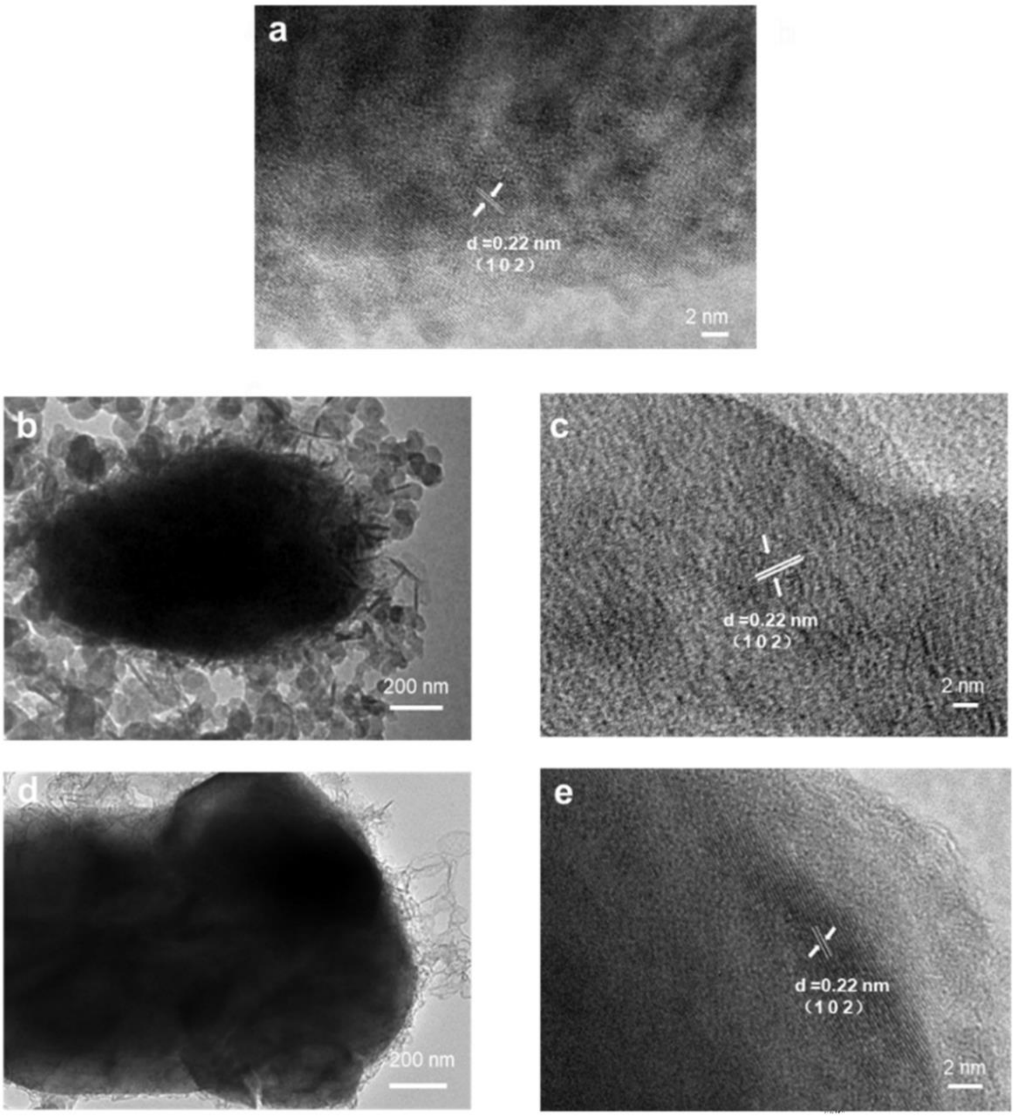

Figure S19 TEM images of discharge products on FeMoO-600 electrode after discharged to 600 mAh g^{-1} at 200 mA g^{-1} in (a) half-sealed condition, (b–c) open air, and (d–e) O_2.

Theoretical calculations

The adsorption energies (E_{ad}) were calculated using the formula:

$$E_{ad} = E_{sub-ads} - E_{sub} - E_{mol}$$

where $E_{sub\text{-}ads}$, E_{sub}, and E_{mol} are the total energy of the adsorption system, slab model, and adsorbed molecules, respectively.

The reaction free energy of each reaction coordinate was calculated by the equation:

$$\Delta G = E - E_0 + \Delta n_{Li} (\mu_{Li} - eU) + \Delta n_{O_2} \mu_{O_2}$$

E and E_0 represent the total energy of the system at specific and initial steps. Δn_{Li} and Δn_{O_2} are the adsorbed/desorbed numbers of Li and O_2 for each step. μ_{Li} and μ_{O_2} are the chemical potentials of Li and O_2. U is the electromotive force corresponding to the discharging, equilibrium, and charging voltage.

The calculated overpotential of ORR and OER processes were defined as

$$\eta_{ORR/OER} = | U_{DC/C} - U_0 |$$

and total potential was

$$\eta_{TOT} = \eta_{ORR} + \eta_{OER}$$

U_{DC} and U_C are the discharge and charge potential which drives all ORR or OER steps energetically downhill, respectively. U_0 is the equilibrium potential, making the ORR/OER process occur spontaneously ($\Delta G \leq 0$).

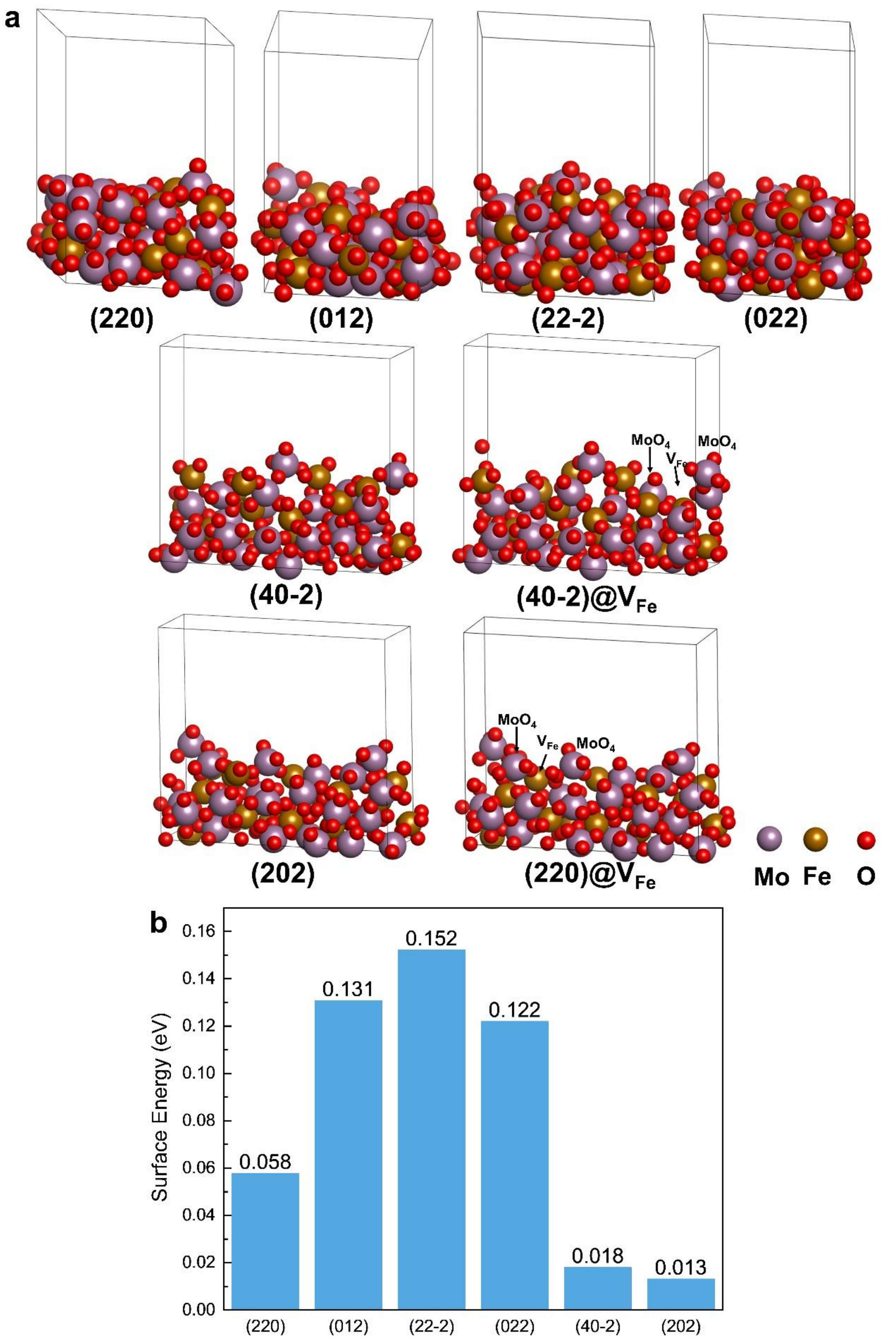

Figure S20 (a) Calculated models of six facets in FeMoO crystal as well as two main facets containing Fe vacancy; (b) calculated surface energy of six crystal facets in FeMoO crystal.

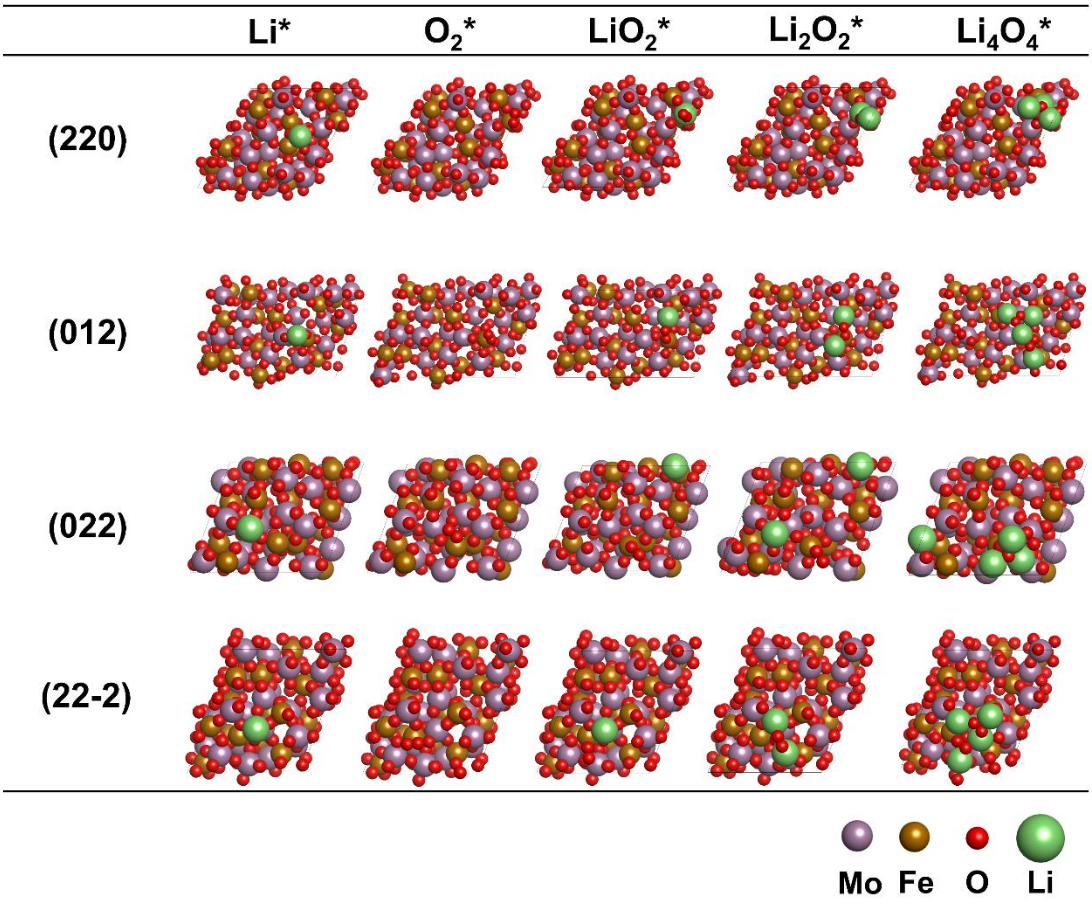

Figure S21 Calculated models of different adsorbates on four facets in FeMoO crystal.

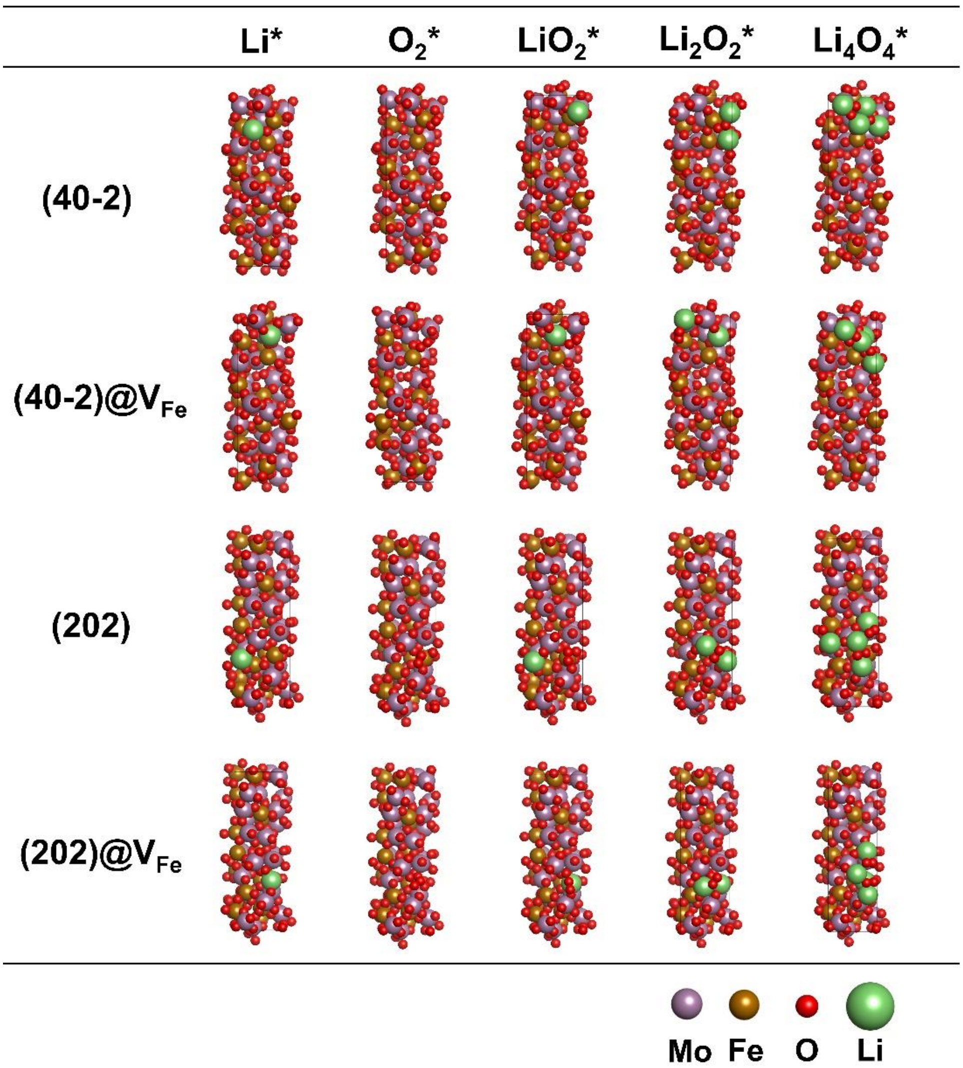

Figure S22 Calculated models of different adsorbates on two main planes as well as those two planes containing Fe vacancy in FeMoO crystal.

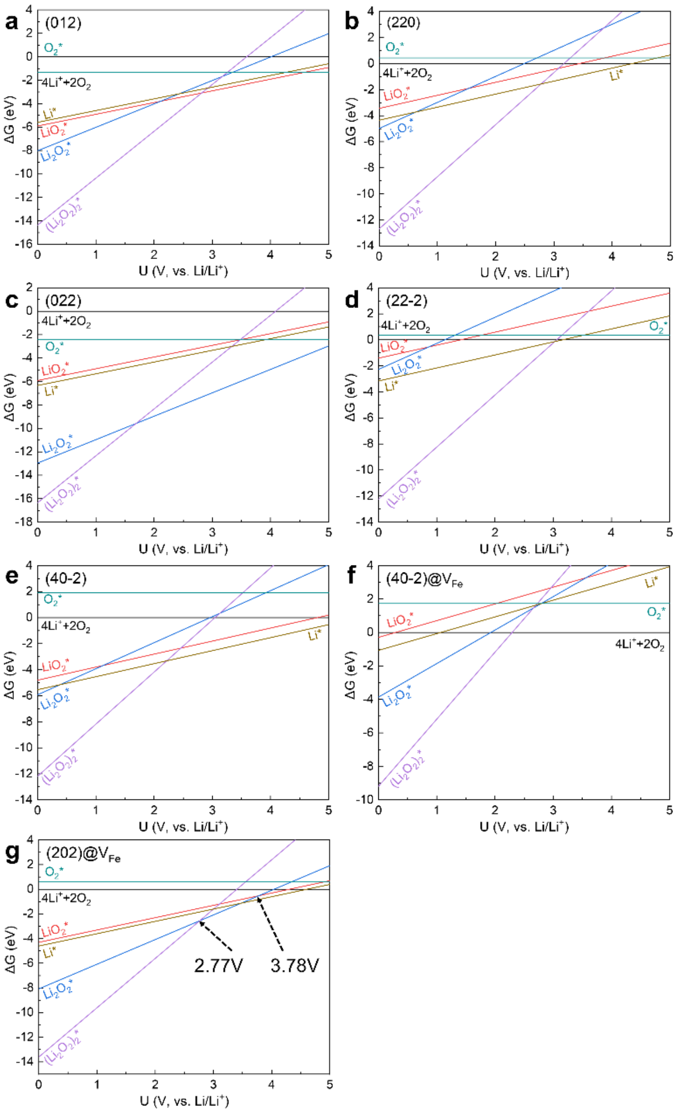

Figure S23 Potential-dependent phase diagram of discharge products for (a) (012), (b) (220), (c) (022), (d) (22-2), (e) (40-2), (f) (40-2) facet with Fe vacancy and (g) (202) with Fe vacancy.

Potential-dependent electrochemical phase diagrams of surface products are of great significance for studying the formation, transformation, and decomposition of products. As shown in Figure S23, the electrochemical phase diagrams of the products for eight different surfaces were calculated. LiO_2 before forms at a high potential for all planes compared to Li_2O_2. However, it is difficult for LiO_2 to exist stably, especially on (22-2) and (40-2)@V_{Fe} surfaces at high potential. $(Li_2O_2)_2$ as a nucleation product has a strong transformation tendency on (012), (220), (22-2), (40-2), (202) surfaces, and has a specific transformation potential on other surfaces, which implies

inhomogeneity of nucleation and polymorphism/amorphism of the products. On the surface of (202), $(Li_2O_2)_2$ has a strong tendency to generate below 2.68V. At the (202) surface with Fe defects, the potential is raised to 2.77 V, but Li_2O_2 is formed as the main product above this voltage. Under the difference between Fe defect and defect-free surface, this difference will lead to a limited or weakened surface nucleation process and the generation of polycrystalline or even amorphous products.

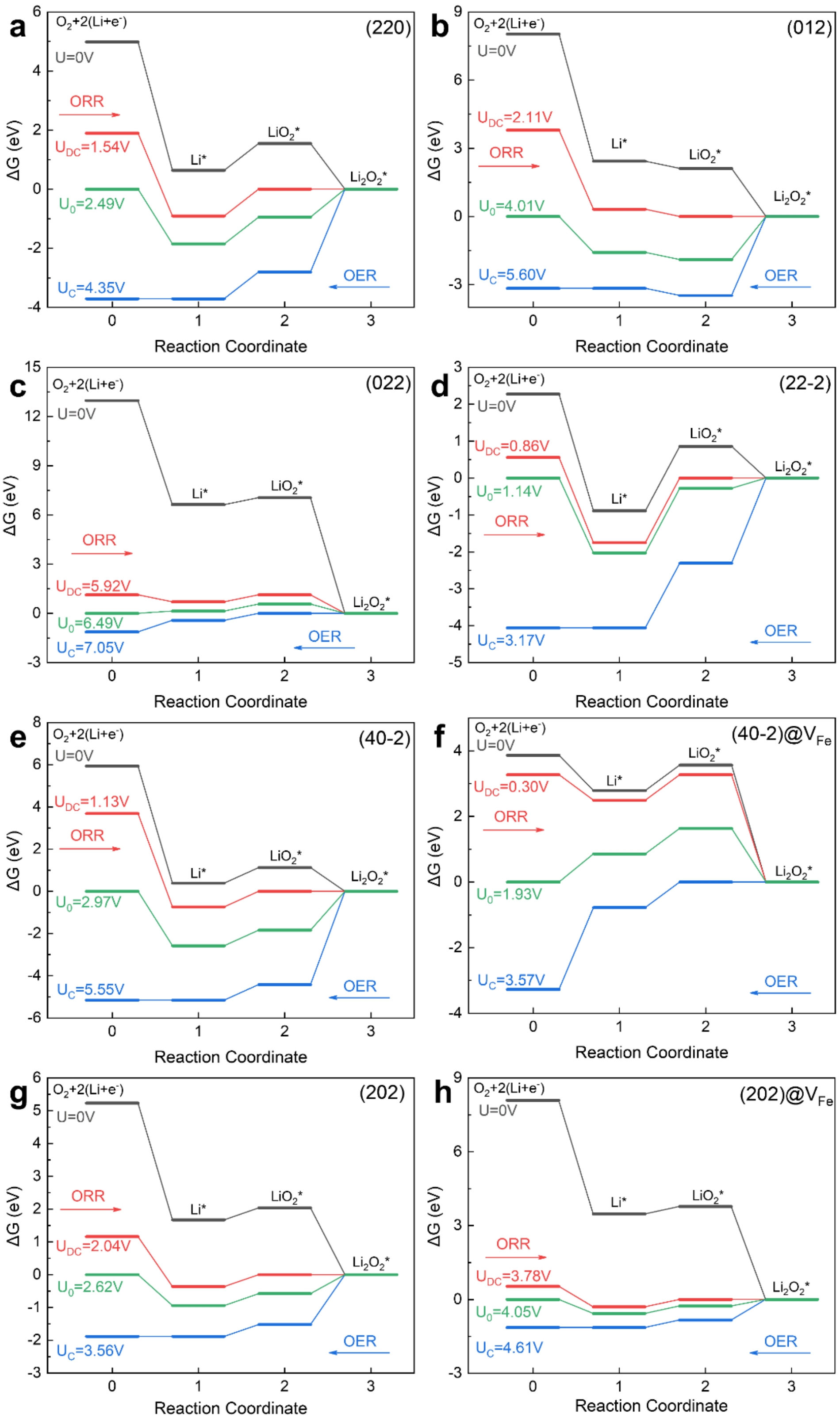

Figure S24 Free energy diagrams based on the designed reaction path with Li_2O_2 as final products.

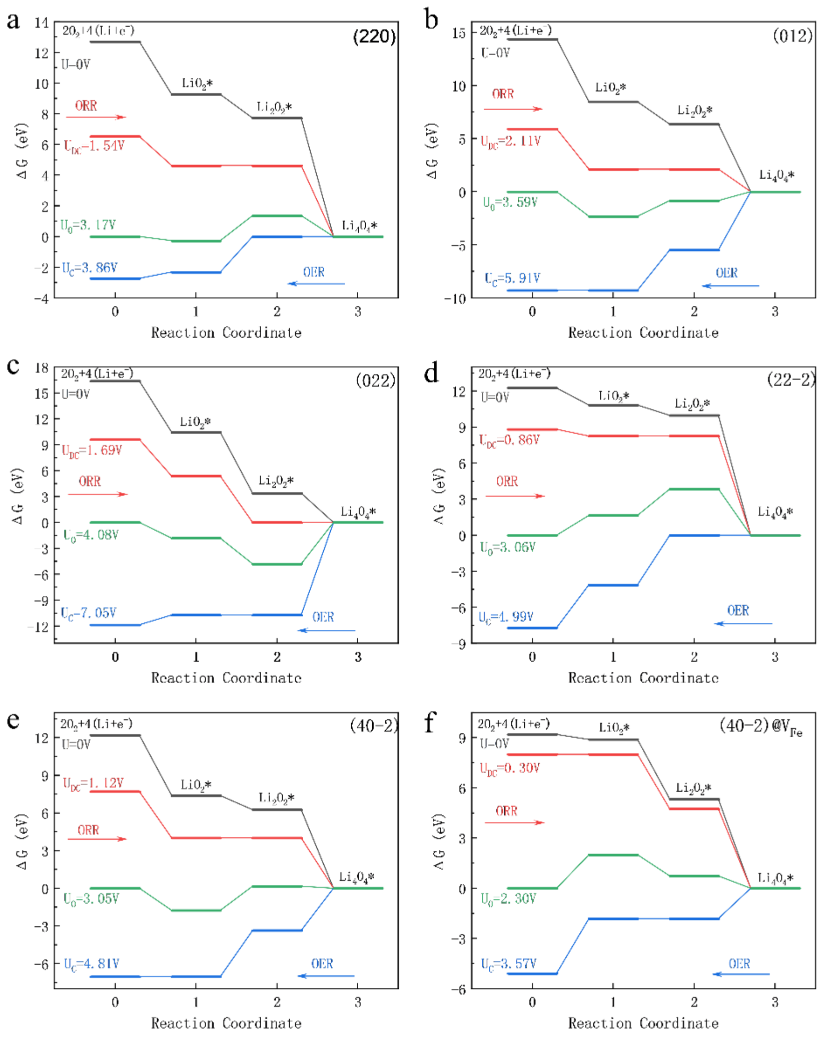

Figure S25 Free energy diagrams based on the designed reaction path with Li_4O_4 as final products.

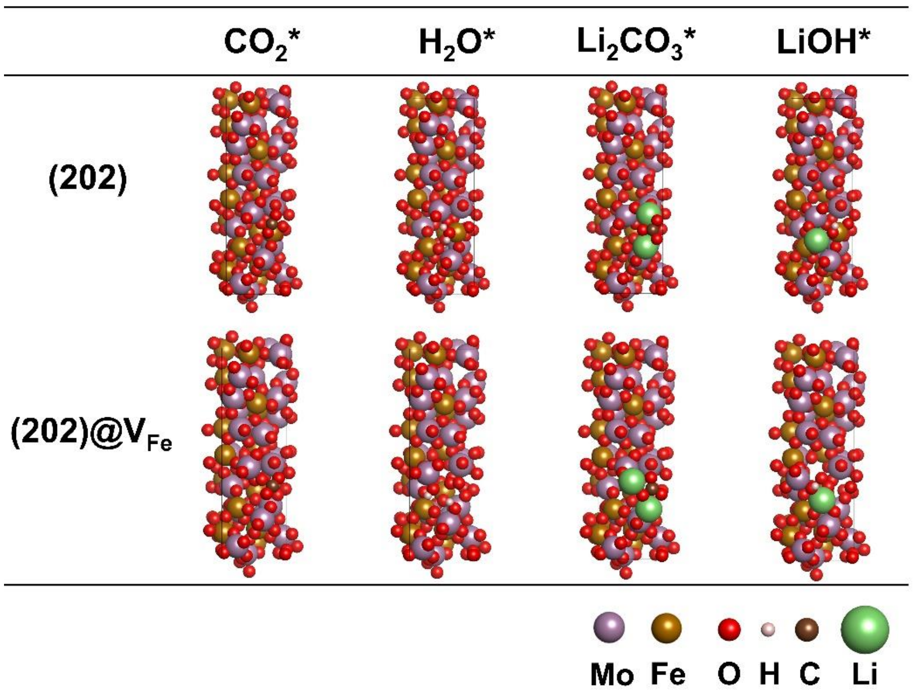

Figure S26 Calculated models of different adsorbates on (202) facet and (202) facet containing Fe vacancy in FeMoO crystal.

Table S1 Comparison of the electrochemical performance of different electrodes using in Lithium-air batteries

Catalysts	Mass/ (mg/cm^{-2})	Electrolyte	Ionic liquid or Redox mediators additive	Circumstances	Oxygen-selective membranes	Current density (mA g-1)	Specific Capacity (mAh g^{-1})	Cycly Number/Limited capacity (mAh g^{-1})	Ref.
MoS_2	0.1	LiTFSI/ DMSO	EMIM-BF4	79%N_2, 21%O_2, 500 ppm. CO_2, RH=45%	-	500	-	550/500 (1100 h)	[11]
Mo_3P/ TEMPO	0.1	LiTFSI/ DMSO	EMIM-BF_4/ TEMPO DBBQ	79%N_2, 21%O_2, 500 ppm. CO_2, RH=45%	-	500	-	1200/500 (2400 h)	[12]
LiI/CNT	<0.1	LiI in Gel electrolyte	LiI	Ambient air RH=5%	LDPE	2000	-	600/1000$_{CNT}$ (600 h)	[13]
CNT-SS	0.05	Zeolite X	LiTFSI in $[C_2C_1im][NTf_2]$	Ambient air	-	500	12020	300/500 (600 h)	[14]
RuO_2/NiO	0.075	LiTFSI/ TEGDME	-	Ambient air RH=65%	-	250	3465	200/500 (400 h)	[15]
MCNTs@MnO_2	0.3~0.5	LiTFEI/ TEGDME	Co^{II}-salen	dry air (21% O_2 , 78% N_2) without CO_2 and H_2O	-	500	13000	300/1000 (1200 h)	[16]

LiI/rGO	0.12	Porous LiI in Gel electrolyte	LiI	Ambient air RH=15%	-	100	3325	400/1000$_{rGO}$ (800 h)	[17]
MoS_2	0.1	LiTFSI/DMSO/EMIM-BF_4	InI_3/InBr3	Dry air		1000		600/1000 (1200h)	[18]
Ru@CNT	1.5	LiTFSI in (DOL)/ (DME) with $LiNO_3$		Cylinder compressed air (fixed water content of≈5600 ppm)	Graphene-Coated Lithium Anodes	200		230/1000 (2300h)<br>50/1000 (500h) (only with catalyst)	[19]
CNT	0.4±0.1	LiTFSI, TEGDME	DBBQ and TEMPO	Ambient air RH=25%	PSS-Li/GO-Li/GF	0.2 mA $/cm^2$		486/0.2 mAh/cm^2 (972h)	[20]
δ-MnO_2@MWCNTs		0.8 M LiTFSI and 0.5 M $LiNO_3$ in PYR_{14}TFSI/DMSO		Air atmosphere (O_2:N_2:H_2O = 4:16:3)		1000		180/1000 (360h)	[21]
MWCNT		LiTFSI/TEGDME/HMDS		Ambient air RH=60%	anode: $Li_6(Si_2O_7)$ cathode: non-polar D4	500		375/1000 (1400h)<br>30/500 (150h) (only with catalyst)	[22]
rGO	0.8-1	gel polymer electrolyte containing LiI and 4 wt% SiO_2	LiI	Ambient air RH=15%	rGO/Li	100	3300	100/500 (1000h)	[23]

MnO	0.4-0.5	$LiClO_4$, (TEGDME, G4) gel electrolyte	Ambient air RH=10-40%	LiEDA	200		235/500 (1175h)<br>134/500 (670h) (only with catalyst)	[24]
graphene–Co_3O_4	0.325	$LiPF_6$/EC/DEC	Ambient air		160		50/200 (125h)	[25]
$NiCo_2O_4$@Ni		$LiPF_6$/EC/DEC	Ambient air		0.5 mA/cm^2		100/1 mAh/cm^2 (400h)	[26]
Fe@$La_{0.6}Sr_{0.4}Co_{0.2}Fe_{0.8}O_3$	1	LiTFSI/DMSO	Ambient air		400	14093	156/500 (390h)	[27]
Co_3O_4@N-HCN	0.4	LiTFSI/TEGDME	80% Ar 20% O2		100	3325	27/500 (270h)	[28]
$NiCo_2O_4$	0.8	LiTFSI/TEGDME	Ambient air (25℃ CO_2 0.03%)		300	8019	40/1000 (266h)	[29]
SWNT/IL CNG	0.6	0.5 M Li[NTf_2] and [C_2C_1im][NTf_2]	Ambient air		200	10730	10/1000 (100h)	[30]
3DOS-Co@Mn@CC	0.63	Gel Electrolyte	Ambient air RH=50%		500	6500	100/500 (200h)	[31]

**This work**	**1.0**	**LiTFSI/TEGDME**	**Ambient air RH=~30%**	**200**	**8000**	**298/600 (1800 h)**
						**289/600 (500 mA g^{-1})**

Table S2 Overpotential calculated from energy diagrams based on the designed reaction path with Li_2O_2 as final products.

	U_{DC}	U_0	U_C	η_{ORR}	η_{OER}	η_{TOT}
**220**	1.54	2.49	4.35	0.95	1.85	2.80
**012**	2.11	4.01	5.60	1.90	1.58	3.48
**022**	5.92	6.49	7.05	0.57	0.57	1.13
**22-2**	0.86	1.14	3.17	0.28	2.03	2.31
**40-2**	1.13	2.97	5.55	1.84	2.58	4.42
**40-2@V_{Fe}**	0.30	1.93	3.57	1.64	1.64	3.27
**202**	2.04	2.62	3.56	0.58	0.94	1.52
**202@V_{Fe}**	3.78	4.05	4.61	0.27	0.57	0.84

Table S3 Overpotential calculated from energy diagrams based on the designed reaction path with Li_4O_4 as final products.

	U_{DC}	U_0	U_C	η_{ORR}	η_{OER}	η_{TOT}
**220**	1.54	3.17	3.86	1.63	0.68	2.31
**012**	2.11	3.59	5.91	1.48	2.32	3.80
**022**	1.69	4.09	7.05	2.40	2.96	5.36
**22-2**	0.86	3.06	4.99	2.20	1.92	4.13
**40-2**	1.13	3.05	4.81	1.92	1.76	3.68
**40-2@V_{Fe}**	0.30	2.30	3.57	2.00	1.27	3.27
**202**	2.04	2.65	3.20	0.61	0.55	1.16
**202@V_{Fe}**	2.77	3.41	4.31	0.64	0.91	1.55

Table S4 The calculated adsorption energy (E_{ad}) of different adsorbates on different planes and corresponding Bader charge analysis result (Q).

	**Q(Li)/e**	**E_{ad}-Li (eV)**	**Q(O_2)/e**	**E_{ad}-O_2 (eV)**	**Q(LiO_2)/e**	**E_{ad}-LiO_2 (eV)**	**Q(Li_2O_2)/e**	**E_{ad}-Li_2O_2 (eV)**
**220**	0.92	-4.35	0.00	0.43	0.92	-3.44	0.92	-4.98
**012**	0.93	-5.60	-0.13	-1.31	0.59	-5.91	1.05	-8.03
**22-2**	0.93	-3.17	0.00	0.36	0.92	-1.42	1.59	-2.28
**022**	0.93	-6.35	-0.13	-2.43	0.42	-5.92	1.23	-12.98
**40-2**	0.92	-5.55	-0.85	1.92	-0.09	-4.81	0.87	-5.93
**40-2@V_{Fe}**	0.92	-1.07	-0.01	1.73	0.90	-0.30	1.64	-3.86
**202**	0.92	-3.56	0.00	1.10	0.92	-3.20	1.22	-5.23
**202@V_{Fe}**	0.93	-4.61	0.05	0.59	0.94	-4.31	1.84	-8.09

Table S5 The adsorption energies of CO_2, H_2O, Li_2CO_3, and LiOH on the (202) facet and (202) facet containing Fe vacancy in FeMoO crystal.

	**Q(CO_2)/e**	**E_{ad}-CO_2 (eV)**	**Q(H_2O)/e**	**E_{ad}-H_2O (eV)**	**Q(Li_2CO_3)/e**	**E_{ad}-Li_2CO_3 (eV)**	**Q(LiOH)/e**	**E_{ad}-LiOH (eV)**
**202**	-2.01	-5.05	0.08	0.00	0.89	-1.85	0.48	-2.22
**202@V_{Fe}**	-2.00	-6.28	0.18	-1.77	0.76	-2.92	0.35	-4.35

Reference：

[1] a)J. Yao, Y. Zhou, J. M. Yan, Q. Jiang, *Advanced Energy Materials* **2021**, 11, 2003701; b)Y.-Y. Wu, Y. Teng, M. Zhang, Z.-P. Deng, Y.-M. Xu, L.-H. Huo, S. Gao, *Sensors and Actuators B: Chemical* **2021**, 329, 129126.

[2] A. A. AlKahlaway, M. A. Betiha, D. Aman, A. M. Rabie, *Environmental Technology & Innovation* **2021**, 22, 101386.

[3] D. Lee, S. Sun, C. Kim, J. Kim, D. Song, K. Lee, J. Kim, T. Song, U. Paik, *Electrochimica Acta* **2022**, 401, 139535.

[4] a)J. L. Hart, K. Hantanasirisakul, A. C. Lang, B. Anasori, D. Pinto, Y. Pivak, J. T. van Omme, S. J. May, Y. Gogotsi, M. L. Taheri, *Nature communications* **2019**, 10, 1; b)L. Liu, Y. Liu, C. Wang, X. Peng, W. Fang, Y. Hou, J. Wang, J. Ye, Y. Wu, *Small Methods* **2022**, 6, 2101280; c)Y. Shang, N. Chen, Y. Li, S. Chen, J. Lai, Y. Huang, W. Qu, F. Wu, R. Chen, *Advanced Materials* **2020**, 32, 2004017.

[5] a)C. Xia, M. Waletzko, L. Chen, K. Peppler, P. J. Klar, J. r. Janek, *ACS applied materials & interfaces* **2014**, 6, 12083; b)G. Zhang, G. Li, J. Wang, H. Tong, J. Wang, Y. Du, S. Sun, F. Dang, *Advanced Energy Materials* **2022**, 12, 2103910.

[6] S. Jiao, X. Ren, R. Cao, M. H. Engelhard, Y. Liu, D. Hu, D. Mei, J. Zheng, W. Zhao, Q. Li, *Nature Energy* **2018**, 3, 739.

[7] B. He, J. Wang, J. Liu, Y. Li, Q. Huang, Y. Hou, G. Li, J. Li, R. Zhang, J. Zhou, *Advanced Energy Materials* **2020**, 10, 1904262.

[8] a)H.-S. Kim, J. B. Cook, H. Lin, J. S. Ko, S. H. Tolbert, V. Ozolins, B. Dunn, *Nature materials* **2017**, 16, 454; b)Y. Wang, Z. Huang, Y. Wang, *Journal of Materials Chemistry A* **2015**, 3, 21314; c)H. Ji, X. Liu, Z. Liu, B. Yan, L. Chen, Y. Xie, C. Liu, W. Hou, G. Yang, *Advanced Functional Materials* **2015**, 25, 1886; d)S. Zhang, G. Wang, J. Jin, L. Zhang, Z. Wen, J. Yang, *Nano Energy* **2017**, 36, 186.

[9] T. C. M. Nepel, C. G. Anchieta, L. F. Cremasco, B. P. Sousa, A. N. Miranda, L. C. C. B. Oliveira, B. A. B. Francisco, J. P. d. O. Júlio, F. C. B. Maia, R. O. Freitas, *Advanced Energy Materials* **2021**, 11, 2101884.

[10] S. Ramesh, C.-W. Liew, *Measurement* **2013**, 46, 1650.

[11] M. Asadi, B. Sayahpour, P. Abbasi, A. T. Ngo, K. Karis, J. R. Jokisaari, C. Liu, B. Narayanan, M. Gerard, P. J. N. Yasaei, **2018**, 555, 502.

[12] A. Kondori, Z. Jiang, M. Esmaeilirad, M. Tamadoni Saray, A. Kakekhani, K. Kucuk, P. Navarro Munoz Delgado, S. Maghsoudipour, J. Hayes, C. S. J. A. M. Johnson, **2020**, 32, 2004028.

[13] L. Wang, J. Pan, Y. Zhang, X. Cheng, L. Liu, H. Peng, *Advanced Materials* **2018**, 30, 1704378.

[14] X. Chi, M. Li, J. Di, P. Bai, L. Song, X. Wang, F. Li, S. Liang, J. Xu, J. J. N. Yu, **2021**, 592, 551.

[15] P. Tan, Z. Wei, W. Shyy, T. Zhao, X. J. E. Zhu, E. Science, **2016**, 9, 1783.

[16] X. Hu, J. Wang, Z. Li, J. Wang, D. H. Gregory, J. J. N. l. Chen, **2017**, 17, 2073.

[17] Z. Guo, C. Li, J. Liu, Y. Wang, Y. J. A. C. Xia, **2017**, 129, 7613.

[18] S. Rastegar, Z. Hemmat, C. Zhang, S. Plunkett, J. Wen, N. Dandu, T. Rojas, L. Majidi, S. N. Misal, A. T. J. A. A. M. Ngo, Interfaces, **2021**, 13, 4915.

[19] Y. Ma, P. Qi, J. Ma, L. Wei, L. Zhao, J. Cheng, Y. Su, Y. Gu, Y. Lian, Y. J. A. S. Peng, **2021**, 8, 2100488.

[20] Q. Han, W. Guo, X. He, T. Liu, X. Liu, X. Zhu, T. Bian, L. Jiang, J. Lu, Y. J. J. Zhao, **2022**, 6, 381.

[21] W. Liang, F. Lian, N. Meng, J. Lu, L. Ma, C.-Z. Zhao, Q. J. E. S. M. Zhang, **2020**, 28, 350.

[22] Y. Wang, M. Yu, T. Zhang, Z. Xue, Y. Ma, H. J. C. S. Sun, Technology, **2022**, 12, 332.

[23] Z. Guo, J. Li, Y. Xia, C. Chen, F. Wang, A. G. Tamirat, Y. Wang, Y. Xia, L. Wang, S. J. J. o. M. C. A. Feng, **2018**, 6, 6022.

[24] X. Lei, X. Liu, W. Ma, Z. Cao, Y. Wang, Y. J. A. C. I. E. Ding, **2018**, 57, 16131.

[25] C. Sun, F. Li, C. Ma, Y. Wang, Y. Ren, W. Yang, Z. Ma, J. Li, Y. Chen, Y. J. J. o. M. C. A. Kim, **2014**, 2, 7188.
[26] L. Li, S.-H. Chai, S. Dai, A. J. E. Manthiram, E. Science, **2014**, 7, 2630.
[27] J. Cheng, Y. Jiang, M. Zhang, Y. Sun, L. Zou, B. Chi, J. Pu, L. J. C. Jian, **2018**, 10, 1635.
[28] J. Wang, M. Fan, W. Tu, K. Chen, Y. Shen, H. J. J. o. A. Zhang, Compounds, **2019**, 777, 944.
[29] L. Zou, Y. Jiang, J. Cheng, Y. Gong, B. Chi, J. Pu, L. J. E. A. Jian, **2016**, 216, 120.
[30] T. Zhang, H. J. A. C. I. E. Zhou, **2012**, 51, 11062.
[31] J. Li, Z. Wang, L. Yang, Y. Liu, Y. Xing, S. Zhang, H. J. A. A. M. Xu, Interfaces, **2021**, 13, 18627.
